# Australian Research on Climate Change and Health Interventions: A Systematic Mapping Review

**DOI:** 10.5694/mja2.70165

**Published:** 2026-03-23

**Authors:** Sotiris Vardoulakis, Luise Kazda, Rebecca Haddock, Alexandra L. Barratt, Forbes McGain, Kinley Wangdi, Enembe Okokon, Daniela Espinoza Oyarce, Gopika Indu, Nigel Goodman, Veronica Matthews, Phoebe Spurrier, Alice McGushin, Georgia Behrens, Madeleine Skellern

**Affiliations:** ^1^ HEAL Global Research Centre, Health Research Institute University of Canberra Canberra Australian Capital Territory Australia; ^2^ Wiser Healthcare Research Collaboration University of Sydney Sydney New South Wales Australia; ^3^ Asthma Australia Canberra Australian Capital Territory Australia; ^4^ University of Melbourne Melbourne Victoria Australia; ^5^ Western Health Melbourne Victoria Australia; ^6^ RMIT University Melbourne Victoria Australia; ^7^ University Centre for Rural Health University of Sydney Lismore New South Wales Australia; ^8^ National Health, Sustainability and Climate Unit, Public Health Division Australian Centre for Disease Control Canberra Australian Capital Territory Australia; ^9^ University of Sydney Sydney New South Wales Australia

**Keywords:** climate change, environment, systematic review

## Abstract

**Objectives:**

To review and thematically map published research on health‐related climate change mitigation or adaptation interventions in Australia.

**Study Design:**

Systematic mapping of published peer‐reviewed research studies and reviews examining outcomes associated with climate change and health interventions in Australia.

**Data Sources:**

MEDLINE, Scopus, Google Scholar, published from 1 January 2008 to 1 March 2024, and manual searches of peer‐reviewed literature.

**Data Synthesis:**

Eighty‐three publications (49 original research, 34 reviews) were included, categorised under four themes: (i) health system decarbonisation (18); (ii) health system adaptation, vulnerability and resilience (24); (iii) health co‐benefits of climate change mitigation (9); and (iv) adaptation outside the health system to protect health from climate impacts (26). Six additional studies spanned several of these themes. Ten decarbonisation studies focused on hospital‐based clinical care interventions. In comparison, adaptation studies focused on interventions in a wider variety of health services and community settings. Twenty publications focused on heat, with fewer publications on other climate‐related hazards (bushfires, floods and droughts). Adaptation interventions largely focused on addressing physical health impacts of climate change, with less attention on psychosocial or mental health impacts. Studies on health co‐benefits of mitigation focused on urban greening, shading, cool materials, healthier diets, carbon pricing of food and Indigenous land management. Across all themes, four studies focused on First Nations peoples. Original studies mainly used survey methods, with three studies employing randomised controlled trials and seven using life cycle assessments. Overall, there was limited evidence of stakeholder engagement.

**Conclusions:**

A sustained increase in research on climate change and health interventions will help realise the vision of ‘healthy, climate‐resilient communities, and a sustainable, resilient, high quality, net zero health system’ of the National Health and Climate Strategy. Evidence from local contexts and priority populations, using interdisciplinary methods and stakeholder engagement, will support action on climate change and health in Australia.

## 1 Introduction

Climate change is undermining the environmental and social determinants of health by disrupting natural systems [1] and is one of the greatest threats to human health and well‐being in Australia and worldwide [2]. Climate change poses complex challenges to the functioning and sustainability of the Australian health system [3]. In addition, the Australian health system contributes about 5%–7% of the total national greenhouse gas emissions [4, 5].

The first National Health and Climate Strategy (the Strategy) set out a whole‐of‐government plan for achieving ‘healthy, climate‐resilient communities, and a sustainable, resilient, high quality, net zero health system’ [6]. The Strategy identifies the importance of coordinated climate and health research and includes research and innovation as one of four enablers to achieve its core objectives.

The Strategy identifies the importance of research that assesses interventions to reduce the impacts of climate change on health and health systems in Australia and to reduce the impact of the Australian health system on the climate. While the number of scientific papers reporting on research related to climate change and human health in Australia and globally has increased significantly in the last 10–15 years [1, 7], most of the existing climate change and health literature focuses on estimating the health impacts of exposure to climate hazards [1]—not on assessing climate change and health interventions.

As such, Action 7.2 of the Strategy committed the Australian Government to commissioning and publishing a scan of Australian research activities pertaining to climate change and health, with a view to informing the prioritisation of future research funding and policy decisions for health adaptation and mitigation [6]. This action was addressed by our 2024 report for the Australian Government Department of Health, Disability and Ageing, albeit with a focus on interventions [8].

Building on the 2024 report, this systematic mapping review aims to provide a thematic overview and gap analysis of research on interventions related to health and climate change adaptation and mitigation in Australia and identify priorities for future research relevant to health practitioners.

## 2 Methods

### 2.1 Selection of Publications

A systematic mapping review methodology was employed to collate, describe and catalogue the relevant Australian scientific evidence. Systematic mapping reviews do not aim to answer a specific question (unlike systematic reviews). Instead, they provide an overview of the literature on a broader topic, helping to identify evidence gaps and formulate policy‐relevant research questions [9]. We followed the Preferred Reporting Items for Systematic reviews and Meta‐Analyses extension for Scoping Reviews (PRISMA‐ScR) guideline (checklist provided in the Supporting Information).

We searched the peer‐reviewed literature published between 1 January 2008 and 1 March 2024 in three bibliographic databases (MEDLINE, Scopus and Google Scholar). Inclusion and exclusion criteria and search terms are provided in Supporting Information (Table S1). Publications were imported into Covidence (https://www.covidence.org) and duplicates removed. Titles and abstracts were independently screened by two reviewers. Potentially relevant articles were retrieved and independently assessed for eligibility by two reviewers, with disagreements resolved at both stages by a third reviewer.

This process was complemented with manual searches of the peer‐reviewed literature cited in the *Medical Journal of Australia*–*Lancet* Countdown reports on health and climate change in Australia [7, 10, 11, 12, 13, 14] and other published reviews that included Australian studies.

### 2.2 Data Extraction and Mapping

Key features of the original research studies were extracted, focusing on the study design and methods, setting and/or population, time period, climate hazard, type and effectiveness of intervention and health or environmental outcomes.

Articles were mapped to four predefined themes drawn from the objectives of the Strategy:

- Theme 1: health system decarbonisation, including studies that explore emissions reduction and sustainability interventions within the health system;
- Theme 2: health system adaptation, vulnerability and resilience, focusing on interventions that support adaptation to climate change and strengthen resilience within the health system;
- Theme 3: health co‐benefits of climate change mitigation, focusing on studies that identify and evaluate the health impacts of emissions reduction; and
- Theme 4: adaptation and resilience interventions outside the health system aiming to protect and improve health from climate change impacts.

**FIGURE 1:**
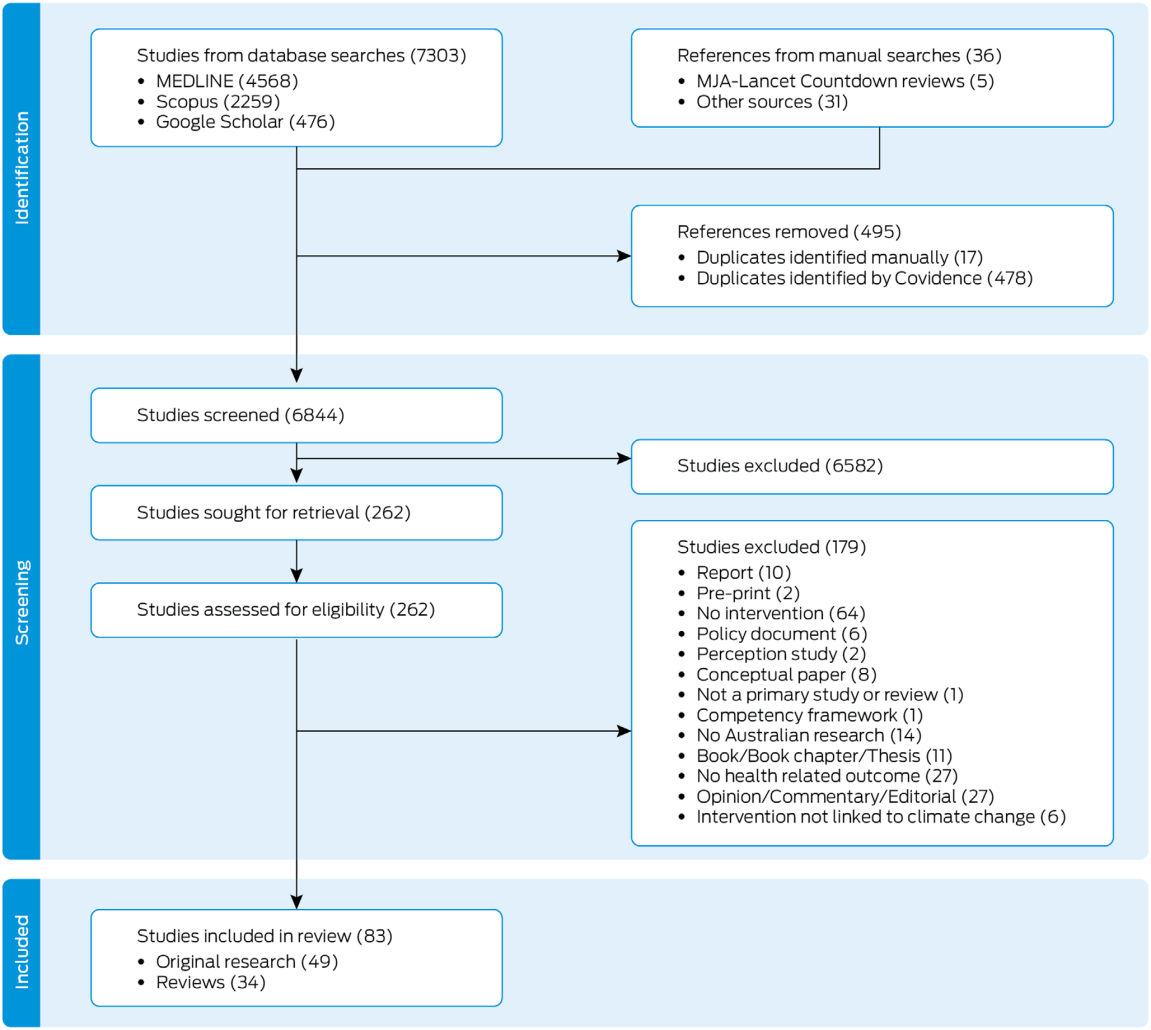
Selection of publications on climate change and health interventions in Australia (PRISMA diagram).

The screening and selection results are presented in Figure 1, and study details by theme in Supporting Information (Tables S2 and S3).

### 2.3 Critical Appraisal

Original research studies were critically appraised using the Quality Assessment with Diverse Studies (QuADS) tool [15] to highlight the strengths and weaknesses of the evidence base. Two reviewers scored the studies independently and an average score was calculated for each study. Original research studies were grouped for similar interventions and the overall quality of studies was appraised for each group.

## 3 Results

Overall, 7303 titles were identified through bibliographic database searches and 36 additional articles through manual searches. After removing duplicates, 6844 titles and abstracts were screened and 262 full‐text articles were retrieved and assessed for eligibility, with 83 of these included in the evidence synthesis. Of these articles, 49 were original research studies and 34 were reviews of the scientific literature. The selected studies were mapped to the four predetermined themes (Tables S2 and S3).

Thirty‐six original research studies were assessed as being of moderate quality, with eight studies being of low quality and five studies of high quality. Across all original studies, evidence of stakeholder engagement in research design and conduct was the quality assessment criterion with the weakest overall score. One‐third of all studies included in this scoping review did not assess the effectiveness of the intervention.

A wide range of research methods were employed in the 49 included original studies, with qualitative or semi‐quantitative study designs involving interviews, focus groups and workshops used in 13 of these studies (Table 1). There were two randomised controlled trials and seven life cycle assessments (studies that model the environmental and financial impacts of a product over its entire life). Of the eligible reviews, 11 were narrative, seven systematic and five scoping reviews.

**TABLE 1:** Overview of original research studies on climate change and health interventions in Australia included in this systematic mapping review, by study design and type of intervention.

Study design and type of intervention	Before and after study	Interviews, focus groups, workshops	Document analysis	Cross‐sectional survey or cohort study	Health impact assessment	Time‐series analysis	Life cycle assessment	Randomised controlled trial	Cost–benefit, environmental, health or socio‐economic analysis	Case report or intervention trial
Environmental benefits in healthcare		Charlesworth and Jamieson [16]								
Single‐use vs. reusable medical equipment							McGain et al. [17] McGain et al. [18] McGain et al. [19] Davis et al. [20]			
Telehealth				Ellis et al. [21]						
Dialysis facilities				Talbot et al. [22]						
Anaesthesia/analgesia	Wyssusek et al. 2022^a^ [23]	Breth‐Petersen et al. [24]					Davies et al. [25]			
Pathology testing	McAlister et al. 2023^a^ [26]						McAlister et al. [27]			
Diagnostic imaging							McAlister et al. [28]			
Healthcare staff training, peer‐support, resilience tool		Knezevic et al. [29] Mohtady Ali et al. [30]								
Hospital campus greening										de Souza et al. [31]
Health promotion		Patrick and Capetola [32]		Patrick and Kingsley [33]						
Heat‐health warnings/behaviours	Nitschke et al.^a^ [34]	Hansen et al. [35]		Williams et al. [36]		Quilty et al. [37]		Nitschke et al. [38]	Williams et al. [39]	
Midwifery care				Kildea et al. [40]						
Primary care			Walker & South East Healthy Communities Partnership [41]							
Public health messaging		Marfori et al. [42]								
Regional collaboration				van Beurden et al. [43]						
First Nations people land management		Schultz et al. [44]		Burgess et al. [45]						
Cool materials, urban greening and shading					Haddad et al. [46] Chen et al. [47] Sadeghi et al. [48]				Qi et al. [49] Santamouris et al.^a^ [50]	
Healthy diets/carbon pricing of food				Ridoutt et al. [51]					Springmann et al. [52]	
Community‐based psychosocial program		O'Donnell et al. [53] Rigby et al. [54] Longman et al. [55] McGill et al. [56]						Cowlishaw et al. [57]		Hart et al. [58]
Heat or smoke refuges										Dufty [59] Wheeler et al. [60]
Facemasks		Seale et al.^a^ [61]								
Mosquito control					Tomerini et al. [62]					
Work health and safety				Varghese et al. [63]						

^a^ Research based on multiple study designs.

**FIGURE 2:**
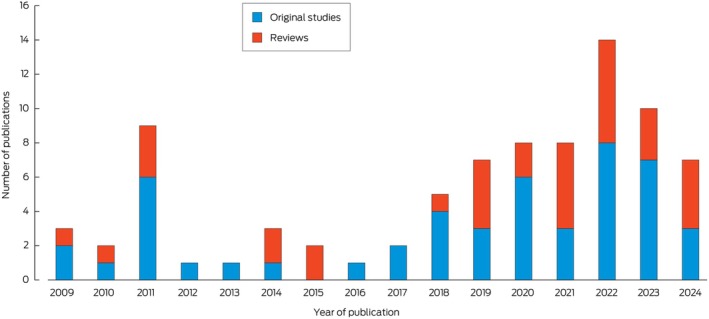
Annual distribution of publications on climate change and health interventions in Australia from 1 January 2008 to 1 March 2024. Figure based on Commonwealth of Australia (Australian Centre for Disease Control) material from the Department of Health, Disability and Ageing systematic mapping report [8], with permission under the Creative Commons Attribution 4.0 International Public License.

Most included studies (30 original studies and 24 reviews) were published in the period 2019–2024, indicating a growing research field (Figure 2).

In terms of geographic distribution, 41 original studies focused on services or populations in specific jurisdictions, including New South Wales (14 publications), Victoria (11 publications), South Australia (6 publications), Queensland (5 publications), Northern Territory (5 publications), Tasmania (2 publications) and Western Australia (1 publication). Three of these studies focused on more than one jurisdiction. There were no studies focused on the Australian Capital Territory. Eight additional studies covered populations or services from across Australia.

### 3.1 Health System Decarbonisation

There were 13 original research studies and five reviews on health system decarbonisation. Seven of the original studies were life cycle assessments. Studies in this theme mainly focused on hospital‐based clinical care interventions aiming to reduce the carbon footprint of specific services. Nine studies were in either anaesthesia/analgesia or pathology/diagnostics, with other fields of health and aged care services being less researched. Original research focused on decarbonising healthcare‐related travel [21], sustainability initiatives for operating rooms [18] and dialysis programmes [22], reducing greenhouse gas emissions from pathology testing [26, 27], ureteroscopy [20], medical imaging [28] and anaesthesia or analgesia practice [17, 19, 23, 24, 25]. One qualitative exploratory study used interviews with healthcare leaders to examine best practice towards low‐carbon models of care [16].

Reviews under this theme covered multiple aspects of healthcare delivery [64], including energy sources, water use, waste management and transport related to health facilities such as healthcare clinics and hospitals [65, 66], and opportunities to make operating theatres more environmentally sustainable [67] or reduce greenhouse gas emissions from nitrous oxide use as an inhaled analgesic [68].

### 3.2 Health System Adaptation, Vulnerability and Resilience

There were 14 original research studies and 10 reviews on climate change adaptation, vulnerability and resilience in the Australian health system. Most studies reported on interventions related to heat, bushfires, floods and climate change more broadly. Specifically, original research studies focused on midwifery group practice [40], planetary health training [69], heat‐health warning systems [34, 36, 38, 39], public health messaging during extreme smoke events [42], health promotion practices and sustainability programmes [32, 33], regional collaboration in rural Australia [43], hospital greening and landscaping [31], primary healthcare principle translation into adaptation activities [41] and other health workforce practice, peer support and well‐being interventions aiming to improve healthcare workers' resilience to climate extremes [29, 30].

Reviews under this theme covered training and education of medical students and health workers in relation to community mental health resilience [70], the use of digital health technologies in healthcare delivery during natural disasters [71] and the impacts of climate events on the health workforce and related workforce responses [72]. Another review focused on interventions in relation to heatwaves, such as heat‐health action plans, which aim to ameliorate the adverse effects of extreme heat on older people [73]. Two reviews covered strategies for delivering mental health services [74] and adaptation measures for childhood asthma [75] in response to climate change. One review discussed more broadly how to prepare health services for climate change in Australia [76], another focused on health promotion principles and interventions for addressing climate change in primary healthcare [77] and two further reviews focused on adaptation strategies to protect population health from environmental extremes and improve management of complex health risks [78, 79].

### 3.3 Health Co‐Benefits of Climate Change Mitigation

There were five original research studies and four reviews on the health benefits (co‐benefits) of climate change mitigation across sectors. Two original research studies focused on the health and well‐being benefits of ‘Caring for Country’ land management activities for First Nations peoples [44, 45]. Another study focused on holistic interventions involving urban greening, shading and cool materials to protect health from extreme heat in a tropical Australian city [46]. Two research studies in this theme assessed healthier diets [51] and carbon pricing of food [52] as a potential intervention towards carbon neutrality.

Reviews in this theme focused on the health benefits of climate change mitigation in relation to active transport [80], policies that can reduce obesity [81], sustainable water and marine ecosystem management [82] and residential energy efficiency [83].

### 3.4 Adaptation Interventions in Sectors Outside the Health System

There were 17 original research studies and nine reviews on adaptation and resilience interventions to protect health from climate change impacts in sectors outside the health system. Six original research studies focused on community‐based psychosocial interventions following multiple disasters [57], including interventions for adults in rural areas affected by droughts and floods [58], supporting children's recovery from bushfires [56], a psychosocial intervention for disaster and trauma survivors [53], adaptation strategies for Aboriginal rural communities affected by prolonged drought [54] and ways to address the mental health and well‐being impacts of recurrent climate extremes in rural communities [55].

Urban heat reduction strategies included urban greening [47, 48], cool materials and shading interventions [49, 50]. Other adaptation studies examined the use of public buildings as shelters during heatwaves [59] or smoke emergencies [60], social and cultural adaptation to heat for First Nations peoples [37] and older persons [35] and related barriers to adaptation. Other original studies explored the use of masks for protection from wildfire smoke among people with pre‐existing respiratory conditions [61], mosquito control interventions [62] and measures for preventing heat‐related injuries and illness in workplaces [63].

Reviews in this theme examined heat adaptation interventions in the home, school and workplace environment, including green infrastructure, shading, water‐sensitive urban design, use of reflective and cool materials, spray systems for evaporative cooling, natural or mechanical ventilation and thermal insulation of buildings and heat‐health warning systems [84, 85]. Two reviews examined risk communication methods in relation to bushfire smoke [86, 87]. A review examined the impacts of climate‐sensitive infectious diseases and related adaptation strategies [88], while another review focused on interventions based on green infrastructure [89]. Three reviews covered multiple adaptation interventions related to mental health [90], women's health [91] and human health more broadly [92] in the context of climate change.

## 4 Discussion

Overall, there is a relatively small but growing number of intervention studies and reviews focused on health‐ and health system‐related mitigation or adaptation interventions in Australia. Original studies mainly used qualitative or semi‐quantitative survey or interview methods, with only two studies employing randomised control trials and seven using comparative life cycle assessments (Table 1).

Original research studies mainly focused on interventions to address the direct physical health impacts (e.g., heat stress, injuries and deaths) of climate change on communities or the carbon footprint of a limited range of clinical services (e.g., anaesthesia, pathology). Eleven studies and reviews considered adaptation interventions to improve mental health outcomes, and community and health workforce resilience to climate change. There were 20 studies and reviews that discussed interventions addressing extreme heat, which were more than the number of studies of interventions directed towards other climate hazards. There was a lack of studies on interventions aiming to build resilience to floods [93].

There were four studies that consider interventions targeting climate‐sensitive infectious diseases, including one original study [62] and three reviews [76, 79, 88]. Bushfire smoke, mainly expressed as fine particulate matter (PM_2.5_), was the subject of two air quality‐related intervention studies [60, 61] and two reviews [86, 87]. However, there was an absence of intervention studies addressing the health impacts of other air pollutants, such as ground‐level ozone and aeroallergens (e.g., pollen), affected by climate change [94, 95]. There were no research studies on interventions in the urban transport and energy sectors, where health co‐benefits from climate change mitigation are likely to be significant [96].

More solution‐focused research is needed to evaluate the effectiveness, acceptability and scalability of health‐related adaptation and mitigation interventions both within and outside the health system. While research is needed across all four themes examined in this review, there is a particular need for more studies of the health co‐benefits of a wide range of mitigation interventions. This requires interdisciplinary research quantifying the potential environmental and health benefits of climate change mitigation interventions across a variety of sectors, such as energy generation, housing, transport, food and agriculture. Health co‐benefit research can inform cross‐sectoral strategies to break down policy silos [97], which can support the implementation of health‐promoting climate change mitigation policies [98].

There is a need for more climate change and health intervention studies with a clear focus on priority populations that are at higher risk of experiencing the adverse health effects of climate change, including First Nations people [99], older people, people who are pregnant, young children, individuals living in rural and remote communities, outdoor workers, culturally and linguistically diverse groups, people living with a disability or chronic health condition and individuals who are socio‐economically disadvantaged [79]. Meaningful engagement and co‐design with priority populations can harness their knowledge and lived experience to develop and deliver more effective adaptation and mitigation interventions. It is important that health inequities are considered as part of the evaluation of climate change mitigation and adaptation interventions [100].

There is a specific need for more whole‐of‐system intervention studies involving practitioners and consumers to support the transition to high value, low carbon healthcare [101]. Low value care increases the greenhouse gas emissions of the health system for little or no health gain [24]. As such, reducing medical overuse can contribute to decarbonising the health system [101], yet there was a notable absence of research on this potential intervention strategy.

Interventional research led or supported by the health workforce needs to be conducted in a wider range of clinical and community settings, including in primary care, aged and allied health services, and urban, regional, rural and remote communities [102]. This will ensure interventions are evaluated in a diverse range of geographic, climatic, socio‐economic and healthcare settings. Key research recommendations are summarised in Box 1.

BOX 1 **Recommendations for future research on climate change and health interventions in Australia.**

- Life cycle assessment studies in different health and aged care settings to identify leverage points for effective decarbonisation interventions. This research can support a transition to low emissions, high‐value care and can inform procurement and reimbursement decisions.
- Health system reform studies to explore innovative interventions to reduce medical overuse and low‐value care and to prioritise preventive health. This could reduce demand for emissions‐intensive hospital‐based care.
- Intervention studies conducted in a diverse range of healthcare settings within different specialty areas throughout primary, secondary and tertiary levels of care, benefiting priority populations (e.g., people living with disability or chronic illness).
- Whole‐of‐system or multi‐component interventions to support the transition to high‐value, low‐emission healthcare and strengthen health system adaptation and resilience, for example, focusing on health workforce resilience as well as service delivery.
- Research on the health co‐benefits of climate change mitigation, examining costs, benefits and barriers and enablers of effective implementation and scale‐up (including priorities for First Nation communities, renewable energy, active transport and sustainable food production and consumption).
- Adaptation research on interventions addressing physical, psychosocial and mental health impacts associated with a diverse range of climate hazards, including (but not limited to) heat, floods, droughts, bushfires, multiple air pollutants and climate‐sensitive infectious diseases.
- Research focused on priority population groups, including First Nations people, people with a chronic illness or disability and culturally and linguistically diverse communities, to assess the role of interventions in building long‐term resilience and adaptive capacity.

### 4.1 Limitations

This scoping review only included peer‐reviewed research from three major bibliographic databases and additional publications identified through manual searches. Relevant studies only available on other research databases or in the grey literature, or studies not explicitly focused on climate change and health interventions, may not have been captured. This review sought to systematically map the existing evidence base and identify evidence gaps, without evaluating the effectiveness of individual interventions.

## 5 Conclusions

There is an urgent need for more research on climate change and health interventions in the Australian health system and communities. Such research will help support progress towards the vision of the National Health and Climate Strategy of ‘healthy, climate‐resilient communities, and a sustainable, resilient, high quality, net zero health system’ [6]. Overall, studies of the health co‐benefits of climate change mitigation are under‐represented and should be prioritised.

Intervention studies need to consider a greater diversity of climate‐related hazards, including heat, bushfires, floods, droughts and air pollution. A more diverse array of research designs as well as systematic reviews of the literature are needed to assess the effectiveness of climate change mitigation and adaptation interventions, including complex whole‐of‐system and multicomponent interventions.

Future research should consider climate‐sensitive infectious diseases, mental health and community and health workforce resilience and should account for variations in health outcomes across Australia. A clearer focus on priority populations, including First Nations people, people with a chronic illness or disability and culturally and linguistically diverse communities, is needed to support health equity in the face of climate change. Primary care, aged care and allied health should be considered—along with clinical specialties beyond anaesthesia, analgesia and pathology.

It is important that new research funding initiatives support and incentivise solution‐focused climate change and health research in Australia. This could be achieved by prioritising interdisciplinary research on place‐based interventions with a clear focus on evaluating effectiveness and implementation, genuine cross‐sectoral collaboration and meaningful stakeholder engagement with community co‐design.

Clinicians and other health professionals can lead or support research that fills the gaps identified for reducing greenhouse gas emissions and adapting to climate change in the broader health sector.

## Author Contributions

Sotiris Vardoulakis contributed to the funding acquisition, conceptualisation, supervision, methodology, formal analysis and writing. Luise Kazda contributed to the formal analysis and writing. Rebecca Haddock contributed to the methodology, formal analysis and editing. Alexandra L. Barratt contributed to the methodology, writing and editing. Forbes McGain contributed to formal analysis and editing. Kinley Wangdi contributed to formal analysis and editing. Enembe Okokon contributed to the methodology, formal analysis and editing. Daniela Espinoza Oyarce contributed to the formal analysis and project administration. Gopika Indu contributed to the formal analysis and editing. Nigel Goodman contributed to the formal analysis and editing. Veronica Matthews contributed to the methodology and editing. Phoebe Spurrier contributed to the editing and project administration. Alice McGushin contributed to the conceptualisation and editing. Georgia Behrens contributed to the conceptualisation and editing. Madeleine Skellern contributed to the conceptualisation, editing and supervision.

## Funding

This scoping review was funded by the Australian Department of Health, Disability and Ageing. We also acknowledge the Healthy Environments And Lives (HEAL) National Research Network, which receives funding from the National Health and Medical Research Council (NHMRC) Special Initiative in Human Health and Environmental Change (Grant No. 2008937).

## Disclosure

Not commissioned; externally peer reviewed.

## Conflicts of Interest

Four co‐authors of this article (Phoebe Spurrier, Alice McGushin, Georgia Behrens, Madeleine Skellern) were employees of the Australian Department of Health, Disability and Ageing, and had involvement in the study conceptualisation and reporting. Sotiris Vardoulakis has received funding from Wellcome Trust, Asthma Australia and Dyson. Forbes McGain has a co‐patent for the McMonty hood (reusable personal protective hood) and a reusable N95 mask (ReResp) and receives royalties for these patents. Sotiris Vardoulakis, Rebecca Haddock, Alexandra L. Barratt and Veronica Matthews are Members of the Australian Government Climate and Health Expert Advisory Group. Sotiris Vardoulakis is Member of the Climate and Health Alliance, the Public Health Association of Australia and the Asthma Australia Research Advisory Committee.

## Supporting information


**Data S1:** mja270165‐sup‐0001‐Supinfo1.pdf.

## Data Availability

This article includes no original data.
