# Supplementary material for: Australian Research on Climate Change and Health Interventions: A Systematic Mapping Review
Source: Med J Aust. 2026 Mar 23;224(3):e70165. doi: 10.5694/mja2.70165 (PMC13006940; doi:10.5694/mja2.70165)
Supplement: Supplementary file 1 — Data S1: mja270165‐sup‐0001‐Supinfo1.pdf. [file MJA2-224-0-s001.pdf]

Supporting Information

Supplementary material

This appendix was part of the submitted manuscript and has been peer reviewed.
It is posted as supplied by the authors.

Appendix to: Vardoulakis S, Kazda L, Haddock R, Barratt AL, McGain F, Wangdi K, Okokon E, Espinoza Oyarce D, Indu G, Goodman N, Matthews V, Spurrier P, McGushin A, Behrens G, Skellern M. Australian Research on Climate Change and Health Interventions: A Systematic Mapping Review. *Med J Aust* 2026; doi: 10.5694/mja2. 70165

.

Australian Research on Climate Change and Health Interventions: A Systematic Mapping Review

Supporting Information

Search Strategy: inclusion and exclusion criteria

We included original research studies (quantitative or qualitative), case studies, systematic, scoping, rapid and narrative reviews published on any of the following topics: (a) health system adaptation and resilience, including healthcare, aged care and public health, (b) health system decarbonisation, including facilities, procurement and healthcare delivery, (c) First Nations' connection to and caring for Country, (d) climate and health education and training, (e) health co-benefits of mitigation in sectors outside healthcare, (f) adaptation in other sectors (including housing, energy, transport, farming and agriculture) addressing the health impacts of climate change.

We excluded: (a) climate change mitigation and adaptation research not specifically focusing on Australia, (b) climate change and health impacts research that did not involve study of proposed or implemented interventions, (c) research on climate change adaptation and mitigation in sectors outside the health system that did not directly refer to health outcomes, (d) health system research that did not directly refer to climate change adaptation, resilience or decarbonisation.

Table S1: Search terms

Search #	Search concept	Search terms
#1	Climate change	(climate OR environment* OR global) NEAR/3 (change OR warming OR sustainab*) OR heat* OR flood* OR drought* OR bushfire* OR wildfire* OR "forest fire*"
#2	Adaption / mitigation / resilience	adaptation OR decarbon* OR emission* OR energy OR green* OR mitigat* OR resilien* OR vulnerab*
#3	Strategies / policies / interventions	"case stud*" OR intervention* OR intelligence OR measure* OR polic* OR promotion OR protection OR standard* OR strateg* OR surveillance OR tracking
#4	Health & wellbeing	"aged care" OR behav* OR communit* OR environment* OR health OR disease OR wellbeing OR "health equit*" OR "health inequit*" OR "health equalit*" OR "health inequalit* OR "health care" OR hospital OR "primary care" OR co-benefit*
#5	Australian setting	Australia* OR "Australian Capital Territory" OR "New

		South Wales" OR "Northern Territory" OR Queensland* OR "South Australia*" OR Tasmania* OR Victoria* OR "Western Australia*" OR "First Nation*" OR Aboriginal OR Indigenous OR Torres Strait
#6		#1 AND #2 AND #3 AND #4 AND #5
		Date limiter: 1 January 2008 – 1 March 2024

Table S2: Original research studies

Reference	Study type & Climate impact	Site(s) & Setting	Aim(s)	Intervention(s) / Outcome(s)	Key finding(s) & Effectiveness
Theme 1: Health system decarbonisation					
Breth-Petersen et al., 2024 [1]	*Study type*: Interviews *Climate impact*: NA	*Site(s):* Four public hospitals in Western Sydney, Australia *Setting:* Hospital	*Aim(s)*: Understand anaesthetists' perspectives on the carbon footprint of anaesthesia and views on shifting towards more environmentally sustainable options.	*Intervention(s):* Evidence-based clinical behaviour change techniques (BCTs) to promote sustainable practice in anaesthesia. *Outcome:* Carbon emissions	*Key finding(s):* Barriers to the wider use of greener anaesthetic agents were identified across all components of the capability, opportunity, motivation and behaviour (COM-B) model: capability (clinician skills and experience, uncertainty around state of the evidence); opportunity (social and professional norms, time and resource pressures); and motivational factors (personal beliefs, habitual clinical behaviour). A sense of responsibility and guilt was reported by several participants as a key motivation for selecting lower emission alternatives. *Effectiveness:* Effective
Charlesworth & Jamieson, 2019 [2]	*Study type*: Semi-structured, in-depth interviews *Climate impact*: NA	*Site(s)*: Australia, UK, USA, NZ *Setting*: Healthcare	*Aim(s)*: Seek healthcare thought leaders' views about a future environmentally sustainable health system.	*Intervention(s):* Adopting an anticipatory approach to healthcare using predictive analytics and using the size and influence of the health sector to effect wider health and environmental benefits. *Outcome(s):* Carbon footprint through low-carbon models of care	*Key finding(s)*: Information communication technology (ICT) was identified as a key enabler. Traditional 'green' initiatives will be insufficient to decarbonise health systems. An anticipatory approach using predictive analytics, adopting anchor institution strategies and using the size and influence of the health sector to effect wider societal change may be important in a low-carbon system of care. *Effectiveness*: Not assessed
Davies et al., 2024 [3]	*Study type*: Retrospective Life Cycle Assessment: comparison of oral vs intravenous (IV) paracetamol *Climate impact:* NA	*Site(s):* USA, UK, and Australian hospitals (10 hospitals from Australia) *Setting:* Hospital	*Aim(s)*: Estimate greenhouse gasses (GHG) emissions associated with intravenous (IV) and oral formulations of paracetamol used in the perioperative period.	*Intervention(s):* Oral paracetamol for intravenous paracetamol in perioperative period. *Outcome(s):* Lifecycle GHG – carbon dioxide equivalent (CO_2e) emissions	*Key finding(s):* Intravenous paracetamol has 12-fold greater life-cycle carbon emissions than the oral tablet form. Glass vials have higher greenhouse gas emissions than plastic vials. *Effectiveness:* Effective
Davis et al., 2018 [4]	*Study type*: Life Cycle Assessment: evaluation of financial and environmental costs *Climate impact*: NA	*Site(s):* Austin Hospital, Melbourne, Australia *Setting:* Hospital	*Aim(s)*: Evaluate and compare the environmental impact of single-use flexible ureteroscopes with reusable flexible ureteroscopes.	*Intervention(s):* Single-use and re-usable ureteroscope *Outcome(s):* Carbon footprint of the lifecycle	*Key finding(s):* The environmental costs of single-use and reusable flexible ureteroscopes were comparable. *Effectiveness:* Inconclusive

Ellis et al., 2013 [5]	*Study type*:<br>Cross-sectional survey<br>*Climate impact*:<br>NA	*Site(s)*:<br>King Island, Tasmania, Australia<br>*Setting*:<br>Healthcare	*Aim(s)*:<br>Quantify the travel conducted by people from one rural area in Australia to access health care, and to calculate the associated carbon emissions.	*Intervention(s):*<br>Telehealth<br>*Outcome(s):*<br>GHG emissions (CO_2e)	*Key finding(s)*:<br>The 134.64 t CO_2e associated with accessing health care (511 health care travel events) for the 12 months of the study period for the participants of King Island represents, in offsets, growing 20 trees for 30 years.<br>*Effectiveness*:<br>Not assessed
McAlister et al., 2020 [6]	*Study type*:<br>Prospective Life Cycle Assessment<br>*Climate impact*:<br>NA	*Site(s)*:<br>Austin Hospital and Sunshine Hospital, Melbourne, Australia<br>*Setting*:<br>Hospital	*Aim(s)*:<br>Estimate the carbon footprint of five common hospital pathology tests: full blood examination; urea and electrolyte levels; coagulation profile; C-reactive protein concentration; and arterial blood gases.	*Intervention(s):*<br>Reducing unnecessary pathology tests.<br>*Outcome(s):*<br>GHG footprint – CO_2e emissions	*Key finding(s)*:<br>Opportunities to reduce the carbon footprint of pathology testing are limited. The carbon footprint of common pathology tests was dominated by those of sample collection and phlebotomy. The greatest environmental benefit can be achieved by reducing unnecessary testing.<br>*Effectiveness*:<br>Not assessed
McAlister et al., 2022 [7]	*Study type*:<br>Prospective Life Cycle Assessment<br>*Climate impact*:<br>NA	*Site(s)*:<br>St George Hospital, Sydney and Footscray Hospital, Melbourne, Australia<br>*Setting*:<br>Hospital	*Aim(s)*:<br>Estimate the carbon footprint of five common imaging modalities within an Australian public hospital setting: computerised tomography (CT), magnetic resonance imaging (MRI), ultrasound (US), chest X-ray (CXR), and mobile CXR (MCXR).	*Intervention(s):*<br>Reducing unnecessary medical imaging or ordering low-impact imaging, reducing standby of scanners, and increasing utilisation rates.<br>*Outcome(s):*<br>GHG footprint – CO_2e emissions	*Key finding(s)*:<br>Magnetic resonance imaging (MRI) and computerised tomography (CT) have large carbon footprints compared to the “traditional” imaging modalities of X-rays and ultrasound. The large carbon footprints of MRI and CT are mostly due to electricity use, and in particular, their standby power use.<br>*Effectiveness*:<br>Not assessed
McAlister et al., 2023 [8]	*Study type*:<br>Comparison of pathology billing records (before & after)<br>*Climate impact*:<br>Carbon emission	*Site(s):*<br>St George Hospital, Sydney, Australia<br>*Setting:*<br>Hospital	*Aim(s)*:<br>Measure the impact of an intervention to reduce unnecessary testing on pathology collections and associated carbon emissions and pathology costs.	*Intervention(s):*<br>Policy to limit pathology testing to Mondays and Thursdays, and posters promoting the policy change (e.g. ‘Twice weekly ordering’ and ‘More is not always better’).<br>*Outcome(s):*<br>Carbon emissions (CO_2e) and cost savings	*Key finding(s):*<br>The Division of Medicine contributes to 20% of hospital pathology activity, and if a similar 10% reduction was achieved over the whole hospital, annual savings would be approximately 1300 kg CO_2e, and $500,000. Extrapolated nationally,16 this could lead to savings in the vicinity of 135,000 kg CO_2e and $56 million.<br>*Effectiveness*:<br>While other studies have shown comparable reductions of 11–12% due to interventions reducing unnecessary pathology testing, this study is the first to estimate the associated reduction in greenhouse gas emissions.
McGain et al., 2010 [9]	*Study type*:<br>Life Cycle Assessment: evaluation of financial and environmental costs<br>*Climate impact*:<br>NA	*Site(s):*<br>Western Hospital, Melbourne, Australia<br>*Setting:*<br>Hospital	*Aim(s)*:<br>Test three hypotheses:<br>(1) single-use trays are more expensive than reusable trays;<br>(2) the life cycle of reusable trays produces less CO_2 and consumes less water than single-use trays; and<br>(3) adding two cotton gauzes and a paper towel increases the financial and environmental	*Intervention(s):*<br>Reusable anaesthesia trays<br>*Outcome(s):*<br>CO_2 emissions, financial, energy and water costs	*Key finding(s):*<br>The annual savings for a six-operating-theatre hospital converting from single-use to reusable plastic trays would be at least $2500 and 70,000 litres of water, but only marginal savings in CO_2 emissions.<br>*Effectiveness:*<br>The financial and environmental savings of a hospital converting to reusable trays are important. It seems difficult to justify persisting with single-use drug trays, particularly with added cotton gauze.

			costs.		
McGain et al., 2012 [10]	*Study type:* Life Cycle Assessment: evaluation of financial and environmental costs *Climate impact:* NA	*Site(s):* Western Hospital, Melbourne, Australia *Setting:* Hospital	*Aim(s):* Examine the life cycles of reusable and single-use central venous catheter kits used to aid the insertion of single-use, central venous catheters in operating rooms.	*Intervention(s):* Reusable and single-use central venous catheter kits *Outcome(s):* CO_2 emissions, financial, energy and water costs	*Key finding(s):* The reusable central venous catheter insertion kits were less expensive than were the single-use kits *Effectiveness:* The environmental costs of the reusable kit were considerably greater than those of the single-use kit.
McGain et al., 2017 [11]	*Study type:* Life Cycle Assessment: evaluation of financial and environmental costs *Climate impact:* NA	*Site(s):* Melbourne, Australia *Setting:* Hospital	*Aim(s):* Define the environmental and financial consequences of five scenarios: (1) the current practice at Hospital 1 of using reusable anaesthetic circuits, face masks, ProSeal Laryngeal mask airways (LMAs), and direct and video-laryngoscope blades and handles; (2) changing the practice at Hospital 1 to that occurring at Hospital 2 of using disposable anaesthetic circuits, and single-use face masks, LMAs, and direct laryngoscope blades, retaining reusable direct laryngoscope handles and reusable video-laryngoscopes; (3) replacing all reusable with single-use / disposable anaesthetic equipment; (4) from Scenario 1, replacing only reusable with single-use face masks; (5) from Scenario 1, replacing only reusable with single-use direct laryngoscope blades.	*Intervention(s):* Reusable anaesthetic equipment *Outcome(s):* CO_2 emissions, financial, energy and water costs	*Key finding(s):* For an Australian hospital with six operating rooms, converting from single-use to reusable anaesthetic equipment saved more than AUD $30000 per annum, but increased the CO_2 emissions by almost 10%. The CO_2 offset is highly dependent on the power source mix, while water consumption is greater for reusable equipment. *Effectiveness:* Reusable anaesthetic equipment saved costs, but increased CO_2 emissions and water consumption.
Talbot et al., 2022 [12]	*Study type:* Cross-sectional survey *Climate impact:* NA	*Site(s):* 132 dialysis facilities within Australia and New Zealand *Setting:* Hospital	*Aim(s):* What are the current environmental sustainability practices implemented within dialysis facilities in Australia and New Zealand?	*Intervention(s):* Sustainability practices within dialysis care (environmental strategy; building infrastructure and energy use, water, waste management; transport; procurement practices; paper use; and climate change preparedness). *Outcome(s):*	*Key finding(s):* Environmental sustainability practices, education, and improvements are currently not prioritized in the 132 dialysis facilities. A minority of facilities reported having an environmental sustainability strategy in place (44 of 132). *Effectiveness:* Effectiveness of environmental sustainability practices within dialysis facilities not evaluated - just uptake. Limitations such as compromises in the design of the survey, only 33% of dialysis services in Australia and New Zealand responded, responders were volunteers, and the nature of the survey

				Environmental sustainability practices: (1) culture; (2) building design, infrastructure and energy use; (3) operations.	limited the quantitative data collection.
Wyssusek et al., 2022 [13]	*Study type*: Quality improvement reports (before & after) *Climate impact*: Greenhouse gas emissions	*Site(s):* Department of Anaesthesia and Perioperative Medicine, Royal Brisbane and Women's Hospital, Queensland, Australia *Setting:* Hospital	*Aim(s)*: Assess and evaluate the impact of sustainability interventions on the environmental and financial cost of inhaled anaesthetic gas use, specifically focusing on reducing medical carbon emissions. The research aimed to guide future initiatives and research in reducing carbon emissions from healthcare practice, particularly in the context of anaesthesia.	*Intervention(s):* Behavioural changes (raising awareness through meetings, posters, and presentations; updates on CO_2 reduction achievements and financial savings; use of low fresh gas flows and end-tidal control) and system changes (phasing out desflurane vaporisers; upgrading intravenous pumps and anaesthetic machines; implementing automated control of anaesthetic concentrations; configuring machines to recirculate sampling gas; setting defaults for low gas with volatile anaesthetics) to reduce the environmental impact of volatile anaesthetics. *Outcome(s):* Global warming potential of a greenhouse gas relative to carbon dioxide over a 100-year period (GWP100) and CO_2e emissions	*Key finding(s):* (1) Quality improvement (QI) strategies in anaesthetic practices aimed to reduce carbon emissions through behavioural and system changes; (2) Behavioural changes included raising awareness of environmental impacts, promoting regional anaesthesia and Total Intravenous Anaesthesia (TIVA), and encouraging low fresh gas flows; (3) System changes involved equipment upgrades, automated anaesthetic concentration control, and configuring machines for efficient gas usage; (4) Despite a slight increase in sevoflurane bottle purchases from 1191 to 1525 bottles in 2019, followed by a decrease to 1264 bottles by the end of 2021 (representing a 6.13% overall increase), the study underscored the environmental impact of volatile anaesthetics and stressed the importance of sustainable healthcare practices in reducing carbon emissions. *Effectiveness*: Effective: Key findings included a drastic decrease in desflurane use at the Royal Brisbane and Women's Hospital, with the number of desflurane bottles decreasing significantly from 800 bottles in 2016 to 35 bottles in 2021. Additionally, the study reported a shift towards desflurane-sparing techniques, highlighting an evolution in practice towards more sustainable anaesthetic practices.
Theme 2: Health system adaptation, vulnerability and resilience					
de Souza et al., 2023 [14]	*Study type*: Case study *Climate impact*: Heat	*Site(s):* Royal Darwin Hospital, Northern Territory, Australia *Setting:* Hospital	*Aim(s)*: Outline specific efforts undertaken to create climate-resilient, culturally safe spaces for staff and patients at Royal Darwin Hospital and to examine how successful responses have been developed through a staff-led process.	*Intervention(s):* H3 Project (Healthy Patients, Workforce and Environment), a phased implementation of low-cost, biophilic landscaping within the constraints of legacy campus infrastructure, funding restrictions and seasonal weather conditions. *Outcome(s):* Cooling impacts, biodiversity	*Key finding(s):* Substantial cooling impacts and improved local biodiversity, and hospital campus aesthetics. Biophilic design offers low-cost adaptation of legacy health infrastructure to climate change that promotes biodiversity, wellbeing and a relationship of reciprocal nurturing between campus users and the environment. This case study set in the harsh climatic environment of the Northern Territory provides a platform for improving cultural safety and hospital health outcomes for First Nations Australians, while promoting Indigenous knowledge and leadership in climate adaptation and mitigation efforts in the healthcare system. *Effectiveness*:

				impacts, stakeholder engagement, workforce impacts, wellbeing impact, Indigenous perspectives, active mobility outcomes	Effective
Kildea et al., 2018 [15]	*Study type*:<br>Cohort study<br>*Climate impact*:<br>Flood	*Site(s):*<br>Major tertiary hospital in South Brisbane, Australia<br>*Setting:*<br>Hospital, community	*Aim(s)*:<br>Does midwifery group practice (MGP) care, compared to standard care (SC), protect pregnant women from the mental health impact (anxiety and depression) of flood in the post-partum period?	*Intervention(s):*<br>Continuity of care through the antenatal, intrapartum, and postpartum periods from MGP to SC.<br>*Outcome(s):*<br>Changing levels of objective hardship and subjective stress	*Key finding(s):*<br>Objective flood-related hardship and subjective stress increased with increasing standard care, women in the MGP (continuity) group appeared to be protected to some degree from both the objective and subjective aspects of their flood experiences.<br>*Effectiveness*:<br>Effective
Knezevic et al., 2023 [16]	*Study type*:<br>Exploratory-descriptive qualitative study<br>*Climate impact*:<br>Bushfire, flood	*Site(s)*<br>Illawarra Shoalhaven Local Health District (ISLHD), New South Wales, Australia<br>*Setting:*<br>Local community centre	*Aim(s)*:<br>(1) What were the experiences and perspectives of healthcare staff who participated in the Wellness Warriors intervention?<br>(2) What knowledge and skills did participating staff gain from the intervention?	*Intervention(s):*<br>Wellness Warriors intervention (healthcare staff training to provide emotional and peer support to colleagues in the aftermath of a disaster).<br>*Outcome(s):*<br>Mental health support skills and knowledge	*Key finding(s):*<br>Healthcare staff developed deep listening skills and the ability to connect with others and confront the core of their concerns. The intervention also strengthened the faith of the participants in their leadership.<br>*Effectiveness*:<br>Effective
Marfori et al., 2020 [17]	*Study type*:<br>Interviews<br>*Climate impact*:<br>Wildfires	*Site(s):*<br>Huon Valley region, Hobart, Tasmania, Australia<br>*Setting:*<br>households affected by the 2019 wildfires	*Aim(s)*:<br>(1) Understand the level of concern about the impacts of smoke on well-being.<br>(2) Investigate how information about smoke and health was received and understood.<br>(3) Assess if public health information influenced individual actions and behaviour.<br>(4) Determine the acceptability of using portable High Efficiency Particulate Air (HEPA) cleaners for managing poor indoor air quality during the wildfires.	*Intervention(s):*<br>Public health messaging related to smoke during the 2019 wildfires in Tasmania, and evaluation of effectiveness of HEPA cleaners to improve residential indoor air quality during extreme episodes of air pollution caused by the wildfires.<br>*Outcome(s):*<br>(1) Public health messaging; (2) Information about smoke and health; (3) promotion of portable HEPA cleaners	*Key finding(s):*<br>Social media played a crucial role in disseminating information, but diverse communication channels were needed. Concerns about timeliness and effectiveness of interventions arose led to some individuals relocating before official advice. HEPA cleaners accepted to reduce personal symptoms and seek reassurance.<br>*Effectiveness*:<br>Effective: HEPA cleaners were generally well-accepted by participants and perceived to be a potentially practical intervention during the 2019 wildfire incident in Tasmania.
McLean et al., 2022 [18]	*Study type*:<br>Qualitative analysis<br>*Climate impact*:<br>Climate change	*Site(s):*<br>Bond University, Gold Coast, Australia<br>*Setting:*<br>University (medical program)	*Aim(s)*:<br>Answer the question: Did this planetary health assignment, designed to engage teams of learners to "take action" on a pressing global issue (climate change), meet the intended outcomes?	*Intervention(s):*<br>Educational program (planetary health integrated across a five-year medical curriculum).<br>*Outcome(s):*<br>Planetary health assignments (aligned with	*Key finding(s):*<br>The planetary health assignment, as part of longitudinal planetary health curriculum integration, supports students to not just be global citizens but also planetary citizens, developing the knowledge, skills, values, and attitudes to tackle the climate crisis.<br>*Effectiveness*:<br>Outcomes evaluated via practical implications route, but

				SDG 13-Climate Action)	effectiveness questioned on whether it met the intended outcomes.
Mohtady Ali et al., 2022 [19]	*Study type*: Case-study/interviews *Climate impact*: Disasters	*Site(s):* Gold Coast and Brisbane, Queensland, Australia *Setting:* Hospital	*Aim(s)*: How can hospital managers and decision makers improve Health Care Workers' disaster resilience?	*Intervention(s):* Health Care Workers' (HCWs) disaster education and training regarding disaster planning and preparedness. *Outcome(s):* HCWs disaster education, wellness and needs	*Key finding(s):* The HCWs Resilience Toolkit encouraged mindfulness amongst leaders, managers, and policymakers supporting four priority HCWs needs: wellness, education, resources, and communication. *Effectiveness:* HCWs Resilience Toolkit proposed but not evaluated
Nitschke et al., 2017 [20]	*Study type*: Randomised Controlled Trial *Climate impact*: Heat	*Site(s):* Adelaide, Australia *Setting:* Community	*Aim(s)*: What is the efficacy of heat-health messages, based on known risk factors in South Australia, which are sent out at the beginning of summer and their health impact on older people?	*Intervention(s):* Information pack comprising: (1) information sheet on how to deal with extreme heat conditions; (2) a "Top Tips Heat-Health Card"; (3) the South Australia Health Department's "Extreme Heat Booklet - a guide to coping and staying healthy in the heat"; (4) three South Australia Health advice factsheets *Outcome(s):* Self-reported heat stress	*Key finding(s):* Little difference between intervention and control groups regarding modifying behaviours during heat, but air conditioner use, application of wet cloth to the body, and confidence in adequacy of information needed to beat the heat was significantly higher in the intervention group. Significant reduction in the incidence of heat stress in the intervention group compared to the control group. *Effectiveness*: Effective
Nitschke et al., 2016 [21]	*Study type*: Ecological study (case-series) *Climate impact*: Heat	*Site(s):* Adelaide, Australia *Setting:* Hospital, emergency department, ambulance service	*Aim(s)*: Did the heatwave warning system (HWS) reduce morbidity and mortality during the study period?	*Intervention(s):* HWS activated by the State Emergency Service of Adelaide, on advice by the Bureau of Meteorology *Outcome(s):* Ambulance call-outs, hospital admissions, emergency department presentations, health-specific outcomes, mortality and morbidity outcomes	*Key finding(s):* Significantly lower (59%) cardiac-related call-outs, (30%) renal, and (56%) heat-related emergency presentations in 2014 compared to 2009. Mortality was not reduced in 2014. *Effectiveness*: Effective in reducing morbidity but not mortality.
Patrick & Capetola, 2011 [22]	*Study type*: Individual and group interviews; document evaluation *Climate impact*: Climate change (heatwaves)	*Site(s):* Victoria, Australia *Setting*: Healthcare	*Aim(s)*: What health promotion practice in Victorian health care settings address climate change and sustainability issues?	*Intervention(s):* Five case studies addressing food, transport, heatwaves, women's health, nature, mental health, and resilience. *Outcome(s):* Health promotion practices	*Key finding(s):* To identify ongoing and planned health-sector lead action and initiatives to boost resilience, mitigation of and adaptation to climate change. *Effectiveness*: Not assessed
Patrick &	*Study type*:	*Site(s):*	*Aim(s)*:	*Intervention(s):*	*Key finding(s):*

Kingsley, 2019 [23]	Survey and interviews<br>*Climate impact*:<br>Climate change and environmental degradation	All Australian states and territories<br>*Setting:*<br>NA	(1) What health promotion and sustainability programmes exist in Australia?<br>(2) Do ecological models guide these programmes and their evaluation approaches?<br>(3) What are the barriers and enablers to evaluating such initiatives?	Health promotion and sustainability programmes<br>*Outcome(s):*<br>Health promotion practices (sustainable and healthy food, active transport, energy efficiency, contact with nature and capacity building).	Evaluations mostly conducted in-house by programme staff, but also by research academics and evaluation consultants. Surveys most commonly used for evaluations, followed by qualitative approaches like interviews, case studies and administering focus groups. Enablers of programme evaluation were interest from the community, agency, partners, team; knowledge and awareness of key health and sustainability issues; and programme champions. Barriers were resource constraints and competing priorities.<br>*Effectiveness*:<br>Not assessed
van Beurden et al., 2011 [24]	*Study type*:<br>Survey and meetings<br>*Climate impact*:<br>Climate change	*Site(s):*<br>16 core Sustain Northern Rivers region organisations, New South Wales, Australia<br>*Setting:*<br>Regional communities	*Aim(s)*:<br>How can health promotion teams effectively establish and support regional collaborations among organizations to address climate change, facilitating the emergence of multiple strategies tailored to the specific needs of each region?	*Intervention(s):*<br>Engagement with strategies to improve transport options, food security and energy sustainability.<br>*Outcome(s):*<br>Inter-organisational collaborations	*Key finding(s):*<br>A highly connected network of organisations emerged and rapidly evolved to collaborate for action on climate change between 2005-2009. There were significant improvements in network density, centralisation, clustering and reciprocity, with member organisations collaborating on a broad range of strategies.<br>*Effectiveness*:<br>Health Promotion can play a vital role in fostering regional collaborations to enhance community resilience to impacts of climate change.
Walker & South East Healthy Communities Partnership, 2009 [25]	*Study type*:<br>Document analysis and discussion<br>*Climate impact:*<br>Climate change	*Site(s):*<br>Victoria, Australia<br>*Setting:*<br>Primary health care	*Aim(s)*:<br>Discussion of primary health care principles and how they might translate into coping, adaptation and mitigation activities within the primary health care sector based on predictions for climate change in Victoria.	*Intervention(s):*<br>Storylines: (1) adaptation: rising cost of carbon; (2) climate change: rising temperature and heatwaves; (3) emergency: extreme weather event (flood); (4) mental health: community stress; (5) mitigation: greening health services.<br>*Outcome(s):*<br>Adaptive capacity	*Key finding(s):*<br>Storylines can be used in community education, with health promotion workers and community members jointly developing a storyline as an early step in a community action project.<br>*Effectiveness:*<br>Unclear: primary health care response to climate change requires very little in terms of new kinds of interventions. Rather it is a question of applying familiar interventions to a new problem.
Williams et al., 2019 [26]	*Study type*:<br>Survey<br>*Climate impact*:<br>Heatwaves	*Site(s):*<br>Regional (non-metropolitan) areas in South Australia and Victoria<br>*Setting:*<br>Households	*Aim(s)*:<br>Assess householders' perspectives and responses to heat and heat-health warnings, including their awareness, recall, and behaviours in managing extreme heat events.	*Intervention(s):*<br>Heat-health warnings and their effectiveness in promoting protective behaviours among the public in response to extreme heat events.<br>*Outcome(s):*<br>(1) Heat-health warnings; (2) Heat-health communication efforts	*Key finding(s):*<br>Warnings were viewed as appropriate, but impact on behaviour varied with concerns raised about public complacency if warnings are seen as common sense. Women showed greater concern than men, and older age groups had lower risk perceptions. Continuous evaluation and tailored communication strategies were recommended to enhance effectiveness of heat-health warnings and address public health risks during heatwaves.<br>*Effectiveness*:<br>Inconclusive: warnings were well received, but their effectiveness in driving substantial behavioural changes was not clearly demonstrated.
Williams et al.,	*Study type*:	*Site(s):*	*Aim(s)*:	*Intervention(s):*	*Key finding(s):*

2022 [27]	Cost-benefit analysis (including interviews with key informants)<br>*Climate impact:*<br>Extreme heatwaves	Adelaide, Australia<br>*Setting:*<br>NA	Provide a descriptive cost-benefit evaluation for an Australian heat-health warning system (HHWS) intervention, focusing on the economic implications of the HHWS in South Australia.	South Australian Heat Health Warning System (HHWS) included public heat warnings, health advisories, and targeted support for vulnerable groups during extreme heat events.<br>*Outcome(s):*<br>Benefit-cost ratio (activation costs and attributable health benefits)	Estimated cost for a one-week activation of the HHWS was AU$593,000. Compared to potential savings based on reduced healthcare utilization during extreme heat events, the benefit-cost ratio ranged from 2.0 to 3.3 (cost-effectiveness). HHWSs are crucial and cost-effective public health responses to heatwaves.<br>*Effectiveness:*<br>Effective: HHWS intervention was effective in reducing the health impacts of heatwaves and mitigating the associated risks.
Theme 3: Health co-benefits of climate change mitigation action outside the healthcare system					
Burgess et al., 2009 [28]	*Study type:*<br>Multivariate regression analysis<br>*Climate impact:*<br>NA	*Site(s):*<br>Arnhem Land, Northern Territory, Australia<br>*Setting:*<br>Indigenous community	*Aim(s):*<br>Investigate the associations between participation in caring for country and health outcomes relevant to excess Indigenous morbidity and mortality.	*Intervention(s):*<br>Six core activities in caring for Country: time on Country; burning of annual grasses; gathering of food and medicinal resources; ceremony; protecting sacred areas; and producing artwork.<br>*Outcome(s):*<br>Type 2 diabetes status, cardiovascular disease (CVD) risk, psychological distress	*Key finding(s):*<br>Preliminary empirical epidemiological support for: (1) the Indigenous assertion that caring for country may deliver health gains through social, cultural and behavioural pathways; and (2) Indigenous requests to conduct research on workable solutions based on social and cultural determinants of health.<br>*Effectiveness:*<br>Caring for Country was associated with better nutrition, more frequent physical activity and fewer chronic disease risk factors and diagnoses.
Haddad et al., 2020 [29]	*Study type:*<br>Health Impact Assessment (simulation study)<br>*Climate impact:*<br>Heat	*Site(s):*<br>Darwin, Australia<br>*Setting:*<br>Municipal	*Aim(s):*<br>What are the impacts of urban heat mitigation measures on building cooling energy needs, peak electricity demand and health?	*Intervention(s):*<br>Increased greenery, application of cooling materials on pavements and roofs, water spray system, shading, and green roofs (used individually and in combination) on urban overheating.<br>*Outcome(s):*<br>Ambient heat, electricity use for cooling, hospital admissions, and mortality	*Key finding(s):*<br>Urban greenery reduces the annual cooling load of residential buildings by 2.6%; cool roofs and pavements by 5.8%; combination of greenery, cool roofs and pavements, and urban shading by 7.2%. Application of cool materials reduces the annual excess hospital admissions from 40.14 to 27.51; greenery reduces it to 34.67; and combining solutions reduces it to 24.49 per 100,000 population. Combination of shading, greening, and cooling materials saves 9.66 excess deaths per year per 100,000 population.<br>*Effectiveness:*<br>Effective
Ridoutt et al., 2021 [30]	*Study type:*<br>Survey analysis<br>*Climate impact:*<br>NA	*Site(s):*<br>Australia<br>*Setting:*<br>Communities	*Aim(s):*<br>Assess greenhouse gas emissions associated with Australian adult diets obtained from the Australian Health Survey, and alternative diets consistent with the Australian dietary guidelines.	*Intervention(s):*<br>Recommended diet based on the Australian dietary guidelines<br>*Outcome(s):*<br>GHG emissions (CO_2e) associated with food production	*Key finding(s):*<br>Dietary climate footprints averaged 3.4 kg CO_2e per person per day, with total energy intake explaining around one quarter of the variation. Energy dense and nutrient-poor discretionary foods contributed around one third.<br>*Effectiveness:*<br>A diet consistent with current Australian dietary guidelines had a 42% lower climate footprint.

Schultz et al., 2018 [31]	*Study type*:<br>Focus groups, interviews<br>*Climate impact*:<br>NA	*Site(s):*<br>Northern Territory and Western Australia<br>*Setting:*<br>4 Indigenous communities	*Aim(s)*:<br>How Indigenous Australians in remote regions experience high levels of wellbeing despite poor health statistics, and how services could more effectively enhance both health and wellbeing?	*Intervention(s):*<br>Indigenous land management (ILM)<br>*Outcome(s):*<br>Mental and physical health and wellbeing benefits	*Key finding(s):*<br>ILM provides opportunities for promoting both individual and community health and wellbeing through empowerment, healthier behaviours, use of Indigenous languages and knowledge transmission across generations.<br>*Effectiveness:*<br>Not formally evaluated, but ILM promoted wellbeing through strengthening sense of identity and relationships, empowering people, providing access to traditional foods and physical activity, limiting access to alcohol, and strengthening and promoting collaboration of community organisations.
Springmann et al., 2018 [32]	*Study type*:<br>Economic, environmental and health analyses<br>*Climate impact*:<br>NA	*Site(s):*<br>Australia<br>*Setting:*<br>Communities	*Aim(s)*:<br>Estimate the impact of integrating the price of greenhouse gas emissions into the price of food commodities on dietary and weight-related risk factors and associated disease burden in Australia.	*Intervention(s):*<br>Carbon pricing of food<br>*Outcome(s):*<br>Disease burden (DALYs), greenhouse gas emissions, tax revenues	*Key finding(s):*<br>Carbon pricing of food in Australia could be beneficial for population health, while generating public finance revenues and supporting emission-reduction commitments.<br>*Effectiveness:*<br>Including a price of $23 per tCO_2-eq into the price of food commodities is compatible with public health objectives to reduce diet-related disease mortality in Australia.
Theme 4: Adaptation and resilience interventions to protect health in sectors outside the healthcare system					
Chen et al., 2014 [33]	*Study type*:<br>Health impact assessment (two-scale modelling approach)<br>*Climate Impact:*<br>Indoor heat stress	*Site(s):*<br>Melbourne (5 buildings facing north, east, south and west), Australia<br>*Setting:*<br>5 residential buildings	*Aim(s)*:<br>Investigate the role of urban vegetation in reducing heat related mortality in the city of Melbourne, Australia.	*Intervention(s):*<br>Increase of urban vegetation in Melbourne Central Business District (CBD).<br>*Outcome(s):*<br>Heat related mortality rate	*Key finding(s):*<br>Average seasonal summer temperatures can be reduced in the range of around 0.5 and 2°C if the city were replaced by vegetated suburbs and parklands, respectively. With the limited buildings and local meso-climates investigated in this study, around 5-28% and 37-99% reduction in heat related mortality rate were estimated by doubling the vegetation cover.<br>*Effectiveness*:<br>Not determined
Cowlishaw et al., 2023 [34]	*Study type*:<br>Randomis~~z~~ed Controlled Trial<br>*Climate impact*:<br>Disasters (bushfires, drought)	*Site(s):*<br>Rural or regional Victoria, Australia<br>*Setting:*<br>Disaster affected regions of rural or regional Victoria	*Aim(s)*:<br>Investigate the efficacy of the Skills fOr Life Adjustment and Resilience (SOLAR) programme delivered by trained community members to residents of disaster affected regions in Australia.	*Intervention(s):*<br>SOLAR programme: a brief, trauma-informed, skills-based psychosocial programme that can be delivered by trained lay community members vs 5-week self-help programme (control).<br>*Outcome(s):*<br>Anxiety and depression, Post-Traumatic Stress Disorder (PTSD), anger, insomnia	*Key finding(s):*<br>SOLAR led to significantly lower levels of anxiety and depression, and PTSD symptom severity between pre- and post-intervention, relative to the Self-Help condition, while controlling for scores at intake. These differences were not statistically different at follow-up. The SOLAR programme was associated with large effect size improvements in posttraumatic stress symptoms over time.<br>*Effectiveness*:<br>Moderately effective
Dufty, 2022 [35]	*Study type*:<br>Case study<br>*Climate impact*:<br>Heat	*Site(s):*<br>Blacktown, Sydney, Australia<br>*Setting:*<br>Community	*Aim(s)*:<br>Develop a strategy for enabling Council-owned public amenities to provide refuges from extreme heat for vulnerable residents	*Intervention(s):*<br>Heat refuge strategy<br>*Outcome:*<br>Emergency management	*Key finding(s):*<br>The interviews were part of the stakeholder engagement to craft the heat strategy.<br>*Effectiveness*:<br>Not assessed

Hansen et al., 2011 [36]	*Study type*:<br>Interviews, focus groups<br>*Climate Impact*:<br>Heat	*Site(s)*:<br>Adelaide, Australia<br>*Setting*:<br>Diverse settings	*Aim(s)*:<br>What are the stakeholders' views of factors influencing the ability of older persons to adapt to hot conditions, and what are the barriers to adaptation based on recent experience?	*Intervention(s)*:<br>Heat-health warnings / Heat protective behaviours<br>*Outcome(s)*:<br>Behavioural adaptation	*Key finding(s)*:<br>Susceptibility of older persons to heat was analysed to revolve around: (1) physiological issues;(2) socio-economic issues; (3) psychological issues; (4) adaptive strategies. Socio-economic issues were very influential in shaping behaviours, as were concerns about power costs when using air conditioning. Publicly cooled spaces can provide a cool environment without costs concerns, but can be problematic due to the lack of care facilities and transport to and from the centres in the heat.<br>*Effectiveness*:<br>Not assessed
Hart et al., 2011 [37]	*Study type*:<br>Case report<br>*Climate Impact*:<br>Drought (but model also used for floods, fire, climate change and economic downturn)	*Site(s)*:<br>Rural New South Wales (9 sites), Australia<br>*Setting*:<br>Community (including health, local service networks and partner agencies)	*Aim(s)*:<br>Describe how the Rural Adversity Mental Health Program was introduced in 2007 to raise awareness of drought-related mental health needs and help address these needs in rural and remote New South Wales.	*Intervention(s)*:<br>Rural Adversity Mental Health Program (RAMHP) raising mental health literacy, organising community social events, and disseminating drought related information.<br>*Outcome(s)*:<br>(1) Mental health first aid (MHFA) training; (2) Community mental health and drought information forums; (3) Booklets for rural health and agricultural service providers	*Key finding(s)*:<br>The RAMHP was accepted in communities as a mechanism for enhancing capacity and resilience during prolonged drought. The core program, which was targeted at all communities facing prolonged drought, was readily able to be adapted to the needs of specific groups, such as older farmers, Aboriginal people, and school students. Responding effectively to the mental health implications of prolonged drought requires long-term interventions that can evolve as community needs change, including when drought breaks.<br>*Effectiveness*:<br>Evaluated and effective according to authors; however, evaluation based primarily on immediate feedback rather than measuring medium-term outcomes (in part, due to planning and financial limitations imposed by annual (rather than longer-term) program funding).
Longman et al., 2023 [38]	*Study type*:<br>Online workshops<br>*Climate impact*:<br>Drought, bushfire, and floods	*Site(s)*:<br>Rural New South Wales (3 areas that experienced extreme weather-related events including drought, bushfire, and floods), Australia<br>*Setting*:<br>Rural communities affected by extreme events.	*Aim(s)*:<br>What participants perceived to be effective at building resilience to the mental health impacts of climate change and the necessary components of success of community resilience building	*Intervention(s)*:<br>Community activities that build resilience to the mental health and wellbeing impacts of climate change (i.e. provision of general community-led support; community-focused climate action, including inclusive and democratic resilience and adaptation planning; and collective politically-focused climate action.<br>*Outcome(s)*:<br>Community-led collective action and planning	*Key finding(s)*:<br>Taking action together can build social and relational capital, engender feelings of belonging and increase informal social connectedness, while simultaneously helping communities prepare for the impacts of climate change.<br>*Effectiveness*:<br>Not assessed
McGill et al., 2024 [39]	*Study type*:<br>Surveys and interviews<br>*Climate Impact*:	*Site(s)*:<br>Rural New South Wales, Australia<br>*Setting*:	*Aim(s)*:<br>What is the impact of the Bushfire Recovery Program on children's wellbeing and	*Intervention(s)*:<br>Psychological interventions supporting children's recovery from bushfires.	*Key finding(s)*:<br>High levels of endorsement by children: children learnt to talk to and trust adults, share thoughts and experiences with others, recognise they were not alone in their experiences.

	Bushfire	Schools and pre-schools	resilience?	*Outcome(s):*<br>Support programmes	Parents strongly agreed that their child(ren) knew who to talk to and trusted adults and understood that changes happen in life. The facilitators' ratings were lower than that of parents.<br>*Effectiveness:*<br>The programme was effective and had positive impacts on children in the areas of coping, expression of emotions, well-being, and peer connections.
O'Donnell et al., 2020 [40]	*Study type:*<br>Surveys and interviews<br>*Climate Impact:*<br>2015 Sampson Flat or Pinery bushfires	*Site(s):*<br>South Australia (Country South Australia Primary Health Network (CSAPHN), the Northern Health Network (NHN), and the Australian Red Cross)<br>*Setting:*<br>Emergency services	*Aim(s):*<br>Describe the development and pilot testing of an internationally developed, brief, and scalable psychosocial intervention that targets distress and poor adjustment following disaster and trauma.	*Intervention(s):*<br>The Skills fOr Life Adjustment and Resilience (SOLAR) program, a skills-based intervention, deliverable by community-based or frontline health or disaster workers with little or no formal mental health training, piloted with 15 Australian bushfire survivors.<br>*Outcome(s):*<br>Psychological impacts: (1) Kessler Psychological Distress Scale (K10); (2) PCL-5 – PTSD Checklist for Diagnostic and Statistical Manual of Mental Disorders, Fifth Edition (DSM-5), Impairment; (3) Mini International Neuropsychiatric Interview Plus 7 (MINI Plus 7); (4) Psychological Outcome Profiles instrument (PSYCHLOPS).	*Key finding(s):*<br>This study provides preliminary evidence that the SOLAR program is an accessible, brief, and scalable psychosocial intervention that can be delivered by trained frontline workers, including volunteers, professional, and paraprofessional health or disaster workers. The pilot also provided preliminary evidence that SOLAR is acceptable to disaster survivors in the Australian context, with all participants who were eligible to participate completing all five sessions of SOLAR, and all who responded to open-ended questions.<br>*Effectiveness:*<br>Found that after training, coaches demonstrated improvements in knowledge and confidence in delivering the intervention and were able to implement the intervention in a safe manner that was acceptable to participants, providing support for the feasibility of the intervention. Limitations like lack of gender or culture influenced treatment response, lack of input on the difficulty level from the coaches and the utilisation of traditional formulae for calculation of the Repeated measures effect size estimates (dRM), which can overestimate the magnitude of effects due to unequal variances.
Qi et al., 2021 [41]	*Study type:*<br>Environmental, social, and economic impact assessment and sensitivity analysis<br>*Climate Impact:*<br>Heat	*Site(s):*<br>Leppington, Sydney, Australia<br>*Setting:*<br>Community	*Aim(s):*<br>Develop a model integrating environmental, social, and economic impact assessment and sensitivity analysis, which considers multiple objectives holistically and enables the key planning and design variables for Urban heat mitigation techniques (UHMTs) to be identified.	*Intervention(s):*<br>Applicable urban heat mitigation techniques (greenery and cool materials).<br>*Outcome(s):*<br>Heat-related mortality rate, economic productivity loss, implementation costs	*Key finding(s):*<br>Change of pavement colour from black to white reduced average air temperature, land surface temperature, heat-related mortality, energy bills and productivity loss by 0.65°C, 6.38°C, 4.43%, 4.15%, and 1.27%, respectively, while it increased outdoor thermal comfort index by 0.13°C.<br>*Effectiveness:*<br>This assessment model allows governments and decision makers to identify the weight of multiple objectives based on their policy goals and priorities, ensuring the high performance UHMTs selected will meet their demands.
Quilty et al., 2023 [42]	*Study type:*<br>Environmental epidemiology	*Site(s):*<br>Northern Territory, Australia	*Aim(s):*<br>Compare non-Indigenous and Indigenous societies in a tropical	*Intervention(s):*<br>Behavioural adaptation to heat.	*Key finding(s):*<br>Improved understanding of the holistic impact of urban heat mitigation techniques (UHMTs) on context-based mitigation

	(time-series analysis)<br>*Climate impact*:<br>Heat	*Setting*:<br>Unclear	environment and explore the relative importance of physiological, sociocultural, and technological and infrastructural adaptations to heat.	*Outcome(s)*:<br>Excess mortality attributable to temperature exposure	performance, supporting decision making related to urban heat mitigation and cooling cities and communities.<br>*Effectiveness*:<br>This study suggests that social and cultural adaptations to increasing hot weather are potentially powerful mechanisms for protecting human health.
Rigby et al., 2011 [43]	*Study type*:<br>Focus groups<br>*Climate Impact*:<br>Drought	*Site(s)*:<br>Rural centres across New South Wales, Australia<br>*Setting*:<br>Aboriginal Communities (6 sites over regional New South Wales)	*Aim(s)*:<br>Report the views of Aboriginal communities of how prolonged drought in rural New South Wales has affected their social and emotional well-being, and of possible adaptive strategies.	*Intervention(s)*:<br>Consultative forums were convened across rural New South Wales under the New South Wales Health Rural Adversity Mental Health Program.<br>*Outcome(s)*:<br>Concept maps to group health issues into three themes: impacts on culture, sociodemographic and economic impacts, loss	*Key finding(s)*:<br>The findings of the study reported that drought was affecting Aboriginal well-being in six related ways: damaging traditional culture; skewing the population profile in smaller centres; exacerbating underlying grief and trauma; undermining livelihoods and participation; aggravating socioeconomic disadvantage; and creating a context for behaviour that brings shame to culture.<br>*Effectiveness*:<br>The limitations identified in the study offers a critical analysis. The authors of the study believed that the Senior employees from larger agencies were overrepresented while grassroots-level workers and those from smaller agencies, remote-dwelling people and Aboriginal community participants were underrepresented, limiting the generalisability of the findings.
Sadeghi et al., 2022 [44]	*Study type*:<br>Universal Thermal Comfort Index (UTCI) simulations, and Health impact assessment (HIA)<br>*Climate impact*:<br>Heatwaves	*Site(s)*:<br>Sydney Greater Metropolitan Region (GMR), Australia<br>*Setting*:<br>Urban environment (across 10 weather stations)	*Aim(s)*:<br>Develop an exposure-response method to quantify the impact of urban greening strategies on human heat balance and predict their benefits on population health. Objectives: (1) predict the impact of greening adaptation strategies on urban heat in the Sydney GMR; (2) quantify cooling benefits of greening adaptation strategies on a human heat stress thermal physiology model and demonstrate their spatial variation throughout the Sydney GMR during a typical heatwave episode; (3) estimate the heat-related health impacts of adaptation strategies during a typical heatwave day in the Sydney GMR through HIA methods.	*Intervention(s)*:<br>Three urban greening infrastructure strategies aimed at mitigating urban heat in the Sydney GMR.<br>*Outcome(s)*:<br>Urban greening strategies to reduce heat levels and heat-related mortality	*Key finding(s)*:<br>(1) Developed a Heat Health Impact (HHI) method using Universal Thermal Climate Index (UTCI) to assess green infrastructure benefits; (2) analysed the effect of greening scenarios on urban heat and human physiology; (3) showed that urban greening reduced UTCI by up to 1.7°C, with significant cooling observed in western and coastal areas; (4) health impact up to 11.7 fewer heat-related deaths per day during heatwaves with greening interventions; (5) emphasis on integrating green infrastructure into climate strategies for urban resilience and public health protection.<br>*Effectiveness*:<br>Evaluated and effective; implementation of urban greening infrastructure led to a significant reduction in heat-related mortality. Urban Cooling Effect (UCE) calculated under different greening scenarios showed a notable cooling impact on urban heat levels and human thermal physiology. However, the study was restricted to a single heatwave episode which may not capture the long-term effects of urban greening interventions on population health and heat-related mortality over different seasons or years.
Santamouris et al., 2020 [45]	*Study type*:<br>Experimental and numerical impact	*Site(s)*:<br>Greater Sydney area, specifically the Local	*Aim(s)*:<br>Assess the impact of regional overheating on urban sustainability and quantify	*Intervention(s)*:<br>Eight heat mitigation scenarios involving the use of reflective materials,	*Key finding(s)*:<br>It found that during heatwaves, temperatures in Western Sydney, particularly in Parramatta, rise significantly compared to coastal areas. Mitigation measures such as increasing

	assessment<br>*Climate Impact:*<br>Urban heat island effect (over-heating)	Government Area of the City of Parramatta in Western Sydney, Australia<br>*Setting:*<br>Urban environment	potential sustainability improvements through the implementation of optimized heat mitigation technologies in the City of Parramatta, Western Sydney, Australia	additional greenery, irrigation to enhance evapotranspiration, and various combinations of these measures to mitigate urban overheating.<br>*Outcome(s):*<br>Cooling potential, impact on energy demand, indoor environmental quality, vulnerability, survivability, and heat-related mortality and morbidity in the urban environment	albedo, planting trees, and enhancing evapotranspiration showed promise in reducing ambient temperatures and mitigating urban heat island effects. Implementing these strategies could improve energy demand, indoor environmental quality, and decrease heat-related health issues. The research underscores the importance of sustainable urban planning to address urban overheating and enhance the resilience of cities to climate change, emphasizing proactive measures to mitigate urban heat island effects and improve overall urban sustainability.<br>*Effectiveness:*<br>Effective: The outcomes of the evaluation indicated that the mitigation scenarios, which included various strategies such as increasing albedo, planting additional trees, and enhancing evapotranspiration, showed promising results in reducing ambient temperatures and addressing urban heat island effects. These measures were found to have the potential to improve energy demand, indoor environmental quality, vulnerability, survivability, and heat-related mortality and morbidity in the urban environment.
Seale et al., 2023 [46]	*Study type:*<br>Semi-structured phone interviews<br>*Climate Impact:*<br>Wildfire smoke	*Site(s):*<br>Communities across Australia.<br>*Setting:*<br>Community (residents of bushfire-prone areas)	*Aim(s):*<br>Utilise a qualitative approach to understand the current reality around mask use and the factors influencing people to (or not to) wear a mask during bushfire events.	*Intervention(s):*<br>Use of facemasks<br>*Outcome(s):*<br>Community mask use and behaviour	*Key finding(s):*<br>The interview responses and data analysis helped the authors to identify four themes: “Limited past experiences with masks for bushfires”; “My favourite strategy is avoidance”; “Relying on visual triggers for use”, “We need a kick-start to get people to use masks”.<br>*Effectiveness:*<br>Not assessed
Tomerini et al., 2011 [47]	*Study type:*<br>Health impact assessment based on Ross River Virus (RRV) disease notification data, and survey on mosquito control<br>*Climate Impact:*<br>Mosquito borne disease: Ross River Virus (RRV)	*Site(s):*<br>73 Queensland local governments in sub-tropical coastal; tropical coastal; subtropical inland; and temperate inland regions.<br>*Setting:*<br>Community	*Aim(s):*<br>Investigate the relationship between different mosquito management strategies and the incidence of RRV in 4 climatic regions in Queensland, Australia.	*Intervention(s):*<br>Different programs for mosquito-borne disease control.<br>*Outcome(s):*<br>Mosquito control programs.	*Key finding(s):*<br>The findings indicated that mosquito control is an effective public health intervention to reduce mosquito-borne disease; The long-term RRV disease rates were lower in areas where the mosquito control program included pre-emptive (rather than reactive) surveillance based on an extensive (rather than incomplete) knowledge of mosquito habitats, and where treatment of both saltwater and freshwater habitats (compared to only saltwater habitats, in coastal areas) occurred.<br>*Effectiveness:*<br>Mosquito control is an effective public health intervention to reduce mosquito-borne disease.
Varghese et al., 2020 [48]	*Study type:*<br>Cross-sectional survey<br>*Climate impact:*<br>Heat stress or hot weather	*Site(s):*<br>Nation-wide online survey (Australia)<br>*Setting:*<br>Health and safety representatives	*Aim(s):*<br>(1) Investigate the types of heat-related injuries and their associated risk factors in Australian workplaces during hot weather.	*Intervention(s):*<br>Preventive measures, including a range of control strategies such as education and training, personal protection, administrative	*Key finding(s):*<br>Despite legal requirements for safe thermal work environments, heat stress training availability is limited, with only 35% of representatives reporting its presence. Specific Work Health and Safety (WHS) legislation addressing hot weather work is lacking nationwide, highlighting the need for

			(2) Describe and assess the prevention measures adopted for outdoor and indoor workers in relation to heat-related injuries.<br>(3) Examine the existing levels of training, policies, and guidelines related to heat stress in Australian workplaces.	controls, and engineering controls.<br>*Outcome(s):*<br>(1) preventive measures for heat-related injuries/incidents in the workplace; (2) heat stress training; (3) hot weather policies and guidelines in the workplace, and temperature measures used as an indicator of heat risk.	comprehensive prevention approaches. Barriers to prevention include worker awareness gaps, insufficient training, and organizational issues. Workplaces employ preventive measures like providing Personal Protective Equipment (PPE), sunscreen, and cool drinking water. The study underscores the importance of hot weather policies, heat stress training, and control measures to reduce heat exposure risks. Understanding workplace risk factors is essential for effective prevention strategies. The research stresses the preventable nature of heat-related illnesses and injuries, advocating for enhanced workplace safety standards, education, and resources to safeguard workers from heat stress and associated health issues.<br>*Effectiveness:*<br>Inconclusive: As the study did not directly assess the impact or outcomes of the interventions against heat stress, it is inconclusive to determine the effectiveness of the interventions based on the information available in the paper.
Wheeler et al., 2021 [49]	*Study type:*<br>Intervention trial<br>*Climate Impact:*<br>Extreme air pollution events / bushfires	*Site(s):*<br>Port Macquarie library in New South Wales, Australia<br>*Setting:*<br>Public library	*Aim(s):*<br>(1) Evaluate the potential for a public building to serve as a cleaner indoor air shelter during smoke events.<br>(2) Assess the efficacy of installing HEPA cleaners within a smaller room inside the library area.	*Intervention(s):*<br>Utilizing a large public building (Port Macquarie library) as a cleaner indoor air shelter during episodes of elevated fire smoke pollution. Additionally, portable HEPA cleaners were installed in a smaller room within the library to further improve indoor air quality by reducing $PM_{2.5}$ concentrations.<br>*Outcome(s):*<br>(1) Infiltration efficiency of outdoor $PM_{2.5}$; (2) additional effect of HEPA cleaners on $PM_{2.5}$ concentrations.	*Key finding(s):*<br>Indoor air quality inside the library was significantly cleaner compared to outdoor air, with an average reduction of 70% in outdoor-generated $PM_{2.5}$ concentrations. Additionally, installing HEPA cleaners in a smaller media room within the library further reduced $PM_{2.5}$ levels by 17% compared to the main library.<br>*Effectiveness:*<br>Effective: proved effectiveness of use of public buildings as clean indoor air shelters and the use of HEPA filters to improve indoor air quality during extreme smoke events. The findings suggested that operating appropriately sized HEPA cleaners in indoor spaces can lead to substantial reductions in $PM_{2.5}$ concentrations, providing a cleaner indoor air environment and potentially protecting public health during episodes of elevated smoke emissions.

Table S3: Reviews

Reference	Review type & Climate impact	Australian studies included & Setting	Aim(s)	Intervention(s) / *Outcome(s)*	Key finding(s) & Effectiveness
Medical Journal of Australia (MJA) – Lancet Countdown					
Zhang et al., 2018 [50]	*Study type:* Perspective *Climate impact*: Climate change impacts, exposures, and vulnerability	*Australian studies included:* NA *Setting:* Not defined	*Aim(s):* Understanding of health and wellbeing-related responses to the impacts of climate change.	*Intervention(s):* Climate change mitigation and adaptation *Outcome(s):* Indicators that explore links between health and climate change.	*Key finding(s):* Australia is vulnerable to the impacts of climate change on health, and policy inaction in this regard threatens Australian lives. *Effectiveness:* Not assessed
Beggs et al., 2019 [51]	*Study type:* Perspective *Climate impact*: Wildfire, heatwaves, and flood	*Australian studies included:* Not specified *Setting:* NA	*Aim(s):* Assess the impacts of climate change on Australians' health and evaluate the risks of policy inaction.	*Intervention(s):* Climate change mitigation and adaptation *Outcome(s):* Indicators that explore links between health and climate change.	*Key finding(s):* The report tracks progress on health and climate change in Australia across 31 indicators divided into five broad domains: (1) climate change impacts, exposures and vulnerability; (2) adaptation, planning and resilience for health; (3) mitigation actions and health co-benefits; (4) finance and economics; and (5) public and political engagement. *Effectiveness:* Not assessed
Zhang et al., 2020 [52]	*Study type:* Perspective *Climate impact*: Heatwaves, bushfire, air pollution (airborne particulates), extreme events (e.g., flooding, cyclones, hail, tornadoes)	*Australian studies included:* Not specified *Setting:* NA	*Aim(s):* The report focuses on the Australia's 2019-20 Black Summer and evaluates bushfire adaptation strategies to better equip the communities for future instances.	*Intervention(s):* Climate change mitigation and adaptation *Outcome(s):* Indicators that explore links between health and climate change.	*Key finding(s):* Substantial increases in both fire risk and population exposure to bushfires are having an impact on the health and economy of Australia. As a result of the "Black Summer" bushfires, the monthly airborne particulate matter ($PM_{2.5}$) concentrations in New South Wales and the Australian Capital Territory in December 2019 were the highest of any month in any state or territory over the period 2000–2019 at 26.0 $\mu g/m^3$ and 71.6 $\mu g/m^3$ respectively, and insured economic losses were $2.2 billion. In addition, the study found a 50% increase in scientific publications and a doubling of newspaper articles on the topic in Australia in 2019 compared with 2018. *Effectiveness:* Not assessed
Beggs et al., 2021 [53]	*Study type:* Perspective *Climate impact*: Heat, bushfires, and other indicators	*Australian studies included:* Not specified *Setting:* NA	*Aim(s):* The report provides an update on the full suite of indicators and highlights two new indicators: "Heat impact on physical and sporting activities", and "Bushfire adaptation" to better equip the communities for future instances.	*Intervention(s):* Climate change mitigation and adaptation *Outcome(s):* Indicators that explore links between health and climate change.	*Key finding(s):* The collaboration tracks the links between public health and climate change across about 40 indicators in five domains: (1) climate change impacts, exposures and vulnerability; (2) adaptation, planning and resilience for health; (3) mitigation actions and health co-benefits; (4) economics and finance; and (5) public and political engagement. *Effectiveness:* Not assessed

Beggs et al., 2022 [54]	*Study type:* Perspective *Climate impact:* Wildfire, heatwave, bushfires, rising sea level, and carbon emissions	*Australian studies included:* Not specified *Setting:* National focus or NA	*Aim(s):* The report tracks progress on an extensive suite of indicators.	*Intervention(s):* Climate change mitigation and adaptation *Outcome(s):* Indicators that explore links between health and climate change.	*Key finding(s):* We track progress on an extensive suite of indicators across these five domains, accessing, assessing and presenting the latest data and further refining and developing our analyses. The study tracks progress in various domains related to health and climate change, including (1) climate change impacts, exposures, and vulnerability; (2) adaptation, planning, and resilience for health; (3) mitigation actions and health co-benefits; (4) economics and finance; (5) and public and political engagement. *Effectiveness:* Not assessed
Beggs et al., 2024 [55]	*Study type:* Perspective *Climate impact:* Bushfire, drought, heatwaves	*Australian studies included:* Not specified *Setting:* National focus or NA	*Aim(s):* The report aims to assess the sustainability requirements needed in health care sector of Australia.	*Intervention(s):* Climate change mitigation and adaptation *Outcome(s):* Indicators that explore links between health and climate change.	*Key finding(s):* We track progress on an extensive suite of indicators across these five domains, accessing, assessing and presenting the latest data and further refining and developing our analyses. *Effectiveness:* Not assessed
Theme 1: Health system decarbonisation					
Duindam, 2022 [56]	*Study type:* Narrative review *Climate impact:* Greenhouse gas emissions	*Australian studies included:* Unclear *Setting:* Healthcare clinics	*Aim(s):* Identify progress on the engagement of healthcare clinics and their contribution to the wider sustainability agenda through decarbonising their activities. Review the most important factors identified in the literature to decarbonising healthcare clinics. Produce a practical, evidenced based guide or set of recommendations for healthcare clinics to assist with transitioning their businesses.	*Intervention(s):* Decarbonising healthcare interventions *Outcome(s):* GHG emissions	*Key finding(s):* The common themes that emerged on what healthcare clinics can focus on to reduce their emissions footprint most efficiently are: energy, waste, behaviours/attitudes, and procurement/supply chain. *Effectiveness:* Inconclusive
Liu et al., 2023 [57]	*Study type:* Narrative review *Climate impact:* Greenhouse gas, nitrous oxide	*Australian studies included:* Not specified *Setting:* Hospital, emergency services, primary care, anaesthesia / analgesia	*Aim(s):* How do different prevention strategies aimed at mitigating the effects of nitrous oxide contribute to improving environmental quality and public health?	*Intervention(s):* Nitrous oxide waste gas capture, destruction, oxidation, reducing use, legislation to minimize residual gas in cylinders, education, and awareness programs. *Outcome(s):* Nitrous oxide emissions	*Key finding(s):* In Australia, nitrous oxide is widely used in birthing suites, paediatric and adult operating theatres, and emergency departments, as well as dental, skin care, and veterinary clinics. However, minimal data are available on total nitrous oxide emissions from anaesthesia practices in Australia. *Effectiveness:* Evaluated and effective, as the review provides opportunities to reduce nitrous oxide emissions, mainly by waste destruction, nitrous oxide gap minimization, use reduction, and raising awareness.
McGain &	*Study type:*	*Australian studies*	*Aim(s):*	*Intervention(s):*	*Key finding(s):*

Naylor, 2014 [58]	Systematic review<br>*Climate impact*: Environmental sustainability	*included:*<br>Not specified<br>*Setting:*<br>Hospitals	What hospital environmental sustainability has been studied?	Mitigation of environmental impacts of hospital care<br>*Outcome(s):*<br>Hospital design, energy, water, travel, procured goods, waste, staff behaviour, CO_2 emissions	There remain significant gaps in the evidence base on hospital sustainability.<br>*Effectiveness:*<br>Not assessed
Pencheon et al., 2009 [59]	*Study type:*<br>Narrative review<br>*Climate impact*: Greenhouse gas emissions	*Australian studies included:*<br>Not specified<br>*Setting:*<br>Healthcare sector	*Aim(s):*<br>(1) Explore the role of the healthcare sector, particularly health professionals and organizations, in responding to the challenges posed by climate change; (2) discuss the need for changes in current practices to reduce carbon emissions within the healthcare sector, drawing examples from the National Health Service (NHS) in the United Kingdom and area health services (AHSs) in New South Wales, Australia; and (3) understand how the healthcare sector can contribute to mitigating climate change and promoting sustainability through strategic approaches and initiatives.	*Intervention(s):*<br>(1) Energy efficient upgrades (energy and water-efficient infrastructure); (2) promotion of active transport (transport access guides for cycling paths, walking routes from rail stations, bus stop locations, and bus frequencies).<br>*Outcome(s):*<br>GHG emissions, energy efficiency projects, active transport and sustainability practices	*Key finding(s):*<br>While the study discusses the significance of addressing climate change as a global health threat, it does not provide detailed outcome measures or evaluations of the effectiveness of the interventions discussed. Overall, the review advocates for proactive measures to combat climate change, reduce carbon footprints, and safeguard the health and well-being of populations in the face of environmental challenges.<br>*Effectiveness:*<br>Inconclusive: There is no explicit mention of an evaluation of the effectiveness of the interventions discussed. The focus is on highlighting the response of the healthcare sector to climate change, efforts to reduce carbon emissions, and initiatives to promote sustainability within healthcare facilities. While the review emphasizes the importance of taking action to address the health risks associated with climate change, it does not provide specific details on the evaluation of the effectiveness of the interventions.
Wyssusek et al., 2019 [60]	*Study type:*<br>Narrative review<br>*Climate impact*: NA	*Australian studies included:*<br>84<br>*Setting:*<br>Hospitals - operating theatres	*Aim(s):*<br>(1) Quantify and qualify current operating room (OR) waste;<br>(2) understand existing practices of waste segregation in ORs in Australia, and how these compare with international practices;<br>(3) investigate best practices in waste management initiatives;<br>(4) understand the financial implication of these initiatives; and<br>(5) determine potential barriers to greening initiatives and how these can be improved.	*Intervention(s):*<br>Different environmental initiatives and economic evaluations, for example: energy saving interventions and reprocessing of single us devices, a Lean Six Sigma methodology, Green Operating Room Committee, switching to reusable sharps containers (and many more).<br>*Outcome(s):*<br>Healthcare ecological footprint, waste, and financial costs.	*Key finding(s):*<br>The findings of the study detail the composition of healthcare waste, current and leading OR waste management practices, financial benefits and implication of such strategies and the barriers preventing the implementation of healthcare waste management initiatives. The study believes that the success of an intervention is determined by its benefit to the healthcare system and sustainability over time.<br>*Effectiveness:*<br>Effective in terms of economics (e.g., amount saved per year), exemplified with combination of energy saving interventions and reprocessing of single use devices could save $US 5 billion over 5 years.
Theme 2: Health system adaptation, vulnerability and resilience					
Blashki et al., 2011 [61]	*Study type:*<br>Narrative review<br>*Climate impact*: Heatwaves, fire risk, infectious diseases, poor air quality, and	*Australian studies included:*<br>Not specified<br>*Setting:*<br>Health system	*Aim(s):*<br>Focus on the responses of the Australian health system to health risks from climate change, and in particular how best to prepare health services for predicted health risks from heatwaves,	*Intervention(s):*<br>Health system responses to climate change.<br>*Outcome(s):*<br>General principles for health system adaptation.	*Key finding(s):*<br>Preparing the health system for climate change requires investment in personnel, infrastructure, and coordination. This needs to be done even in the context of uncertainty about the extent and nature of specific climate change effects on health. Key characteristics of a climate change–prepared health system are flexibility, robustness, and

	the mental health impacts		bushfires, infectious diseases, diminished air quality, and the mental health impacts of climate change.		strategically allocated resources. Long term planning will require close collaboration with the non-health sectors as part of a nationwide adaptive response.<br>*Effectiveness:*<br>Not assessed
Crandon et al., 2022 [62]	*Study type:*<br>Narrative review<br>*Climate impact*:<br>Climate change	*Australian studies included:*<br>0 - 4<br>*Setting:*<br>Not specified	*Aim(s):*<br>Provide an overview of the current evidence to inform the response of the mental health sector to climate change.	*Intervention(s):*<br>Community psychological resilience:<br>(1) Community preparedness and response.<br>(2) Sustainable change and advocacy.<br>(3) Education and awareness<br>*Outcome(s):*<br>Non-clinical actions or approaches.	*Key finding(s):*<br>Highlights the prevalence and determinants of mental health issues associated with climate change, climate anxiety in children and adolescents, and the use of psychotropic drugs in relation to heat-related hospitalization risks. Vulnerable populations, such as those with psychosis, dementia, or substance misuse, are particularly susceptible to temperature-related deaths. Emphasis on the importance of psychological resilience and community coping strategies to address climate-related mental health impacts effectively. Overall, it underscores the complex interplay between climate change and mental health, urging targeted interventions and expanded research efforts to mitigate these challenges.<br>*Effectiveness:*<br>Unclear: Narration of how climate change might affect mental health.
Hu et al., 2022 [63]	*Study type:*<br>Systematic review<br>*Climate impact*:<br>Heat, dust storms, forest fires	*Australian studies included:*<br>4<br>*Setting:*<br>Not specified	*Aim(s):*<br>(1) Systematically evaluate the epidemiological evidence on climate change adaptation measures for children with asthma; (2) understand current knowledge gaps; and (3) propose future research directions in this field.	*Intervention(s):*<br>Adaptation measures for childhood asthma (vulnerability assessment, improving ventilation and heating, enhancing community education, developing forecast models and early warning systems).<br>*Outcome(s):*<br>Asthma-related emergency room and hospital admissions	*Key finding(s):*<br>(1) A non-significant trend towards a positive association was observed between forest fires and childhood asthma.<br>(2) Dust storms were associated with increased asthma emergency room visits in children aged ≤ 5 years.<br>(3) Male children and children aged 0-4 and 5–9 years had more emergency room visits during periods of extreme heat.<br>*Effectiveness:*<br>Not assessed
Lokmic-Tomkins et al., 2023 [64]	*Study type:*<br>Narrative review<br>*Climate impact*:<br>Bushfire, flood	*Australian studies included:*<br>None (but studies from Australia mentioned in narrative synthesis)<br>*Setting:*<br>Not specified	*Aim(s):*<br>Examine Digital Health Technologies (DHTs), their advantages and liabilities DHTs confer in the likelihood of climate-related disasters, their prior planning requirements, infrastructural requirements, and vulnerabilities in delivering effective care to the affected populations.	*Intervention(s):*<br>DHTs for supporting healthcare to populations affected by disaster.<br>*Outcome(s):*<br>Responsiveness of DHT supported services to disaster risks and events.	*Key finding(s):*<br>During the Australian bushfires, emergency evacuation instructions sent via digital messaging were effective, but electronic health records (EHRs) were underutilised. During 2022 floods, the Australian EHRs served residents in flood-affected areas in areas such as telehealth appointments, and e-prescribing.<br>*Effectiveness:*<br>Inconclusive: The perceptions of affected community members of DHT effectiveness were not assessed; requirements for optimization of quality healthcare delivery were not assessed.
Palinkas et al.,	*Study type:*	*Australian studies*	*Aim(s):*	*Intervention(s):*	*Key finding(s):*

2020 [65]	Narrative review<br>*Climate impact*:<br>Extreme weather (hurricanes, floods, droughts, wildfires, heatwaves)	*included:*<br>Not specified<br>*Setting:*<br>Not specified	Describe the types and characteristics of mental health services and interventions for the prevention and treatment of mental and behavioural health problems associated with climate change events.	Monitoring and treating mental health problems, strengthening individual and community resilience, training community health workers, conducting inventories of available resources and assessments of at-risk populations, advocacy for mitigation and adaptation programs, risk communication.<br>*Outcome(s):*<br>Strategies for preparedness and response to mental health impacts.	Some of the mental health impacts and services employed in response to these impacts will cut across acute and extreme events, subacute events, and events which permanently alter the physical environment. Some interventions are specific to each type of event. The services which currently target acute and extreme weather events will likely be effective in responding to the mental health consequences of longer duration events.<br>*Effectiveness:*<br>Not assessed
Vu et al., 2019 [66]	*Study type:*<br>Systematic review<br>*Climate impact*:<br>Heatwaves	*Australian studies included:*<br>7<br>*Setting:*<br>Community frontline healthcare services, primary care, and community organizations	*Aim(s):*<br>Identify current measures in mitigating the adverse effects of extreme heat events in older populations. Specifically, examine older people's perceptions and behaviours against existing heatwave prevention measures, categorize and analyse those measures using the Ottawa Charter for Health Promotion framework, and highlight gaps in existing heat action plans.<br>The study also aimed to establish the need for a more coordinated approach to address the potential public health challenges posed by extreme heat events affecting older populations.	*Intervention(s):*<br>Heat Action Plans (HAPs) to mitigate the adverse effects of extreme heat events on older populations (raise awareness, improve vulnerability perception, and promote protective behaviours).<br>*Outcome(s):*<br>(1) Heat Action Plans; (2) Healthcare Provider Training; (3) Enhanced social support and nursing care during heat events; (4) Community-based approaches in improving heat health knowledge and preventive behaviours among older populations.	*Key finding(s):*<br>(1) Older adults were aware of heat risks but often did not perceive themselves as vulnerable; (2) HAPs have reduced mortality and improved behaviours, but confusion remains about specific actions during heatwaves, and causal relationships with outcomes are unclear; (3) more proactive support from healthcare and community personnel is needed to prevent heat-related illnesses among older individuals, enhanced social support and community-based approaches can strengthen resilience; (4) ongoing efforts are essential to improve heat health prevention, better evaluation of HAPs, improved stakeholder coordination, and further research to assess intervention impacts on health outcomes.<br>*Effectiveness:*<br>Inconclusive: HAPs successful in reducing mortality and morbidity rates and improving adaptive behaviours among older populations; however, lack of clear causal relationships between individual components of HAPs and specific health outcomes suggests that further research and refinement in methodology are needed to determine the true effectiveness of these interventions.
Walker et al., 2011 [67]	*Study type:*<br>Systematic review<br>*Climate impact*:<br>Environmental disasters (droughts, storms, and floods); other hazards (heat, water & air	*Australian studies included:*<br>Not specified<br>*Setting:*<br>Not specified	*Aim(s):*<br>Explore the literature where key concepts in primary health care and health promotion are applied to the issue of climate change. The study discussed health promotion principles and intervention strategies for addressing climate change mitigation and adaptation within the primary health care sector.	*Intervention(s):*<br>Health communication, community building, and settings approaches (examples drawn from literature on community resilience and summer heat).<br>*Outcome(s):*<br>(1) Health communication;<br>(2) Community building;<br>(3) Settings approach;<br>(4) Multi-level interventions;	*Key finding(s):*<br>(1) Climate change disproportionately affects vulnerable groups such as low-income individuals, the elderly, Indigenous Australians, and those in poor-quality housing; (2) interventions should promote health equity, climate stabilization, and poverty eradication while addressing specific needs of vulnerable populations; (3) multi-level interventions targeting individuals, households, populations, and communities are essential for greater impact; (4) advocacy actions are crucial for promoting policy changes and community initiatives to address

	pollution, vector-borne and food-borne infectious diseases)			(5) Advocacy actions.	climate change.<br>*Effectiveness:*<br>Inconclusive: No explicit mention of evaluation of the effectiveness of the intervention measures.
Walter et al., 2024 [68]	*Study type:*<br>Integrative review<br>*Climate impact*:<br>Extreme weather events	*Australian studies included:*<br>Not specified<br>*Setting:*<br>Not specified	*Aim(s):*<br>Provide insights into approaches for addressing Climate Change Adaptation (CCA) in the context of public health in Australia.<br>Objectives:<br>(1) Conduct an integrative review to capture a comprehensive understanding of tools, frameworks, guidance material, or methods relevant to CCA and appropriate for preventative public health measures.<br>(2) Explore key themes that can assist in bridging knowledge-action gaps in the context of Australian CCA for public health.	*Intervention(s):*<br>Tools, frameworks, and guidance material suitable for climate change adaptation.<br>*Outcome(s):*<br>(1) National Adaptation Plan;<br>(2) Improving understanding in managing complex health risks;<br>(3) Strengthening the public health system;<br>(4) Building health resilience.	*Key finding(s):*<br>Australia is not adequately prepared to manage the adverse health impacts of climate change, putting the population at risk. The review identified a lack of effective adaptation strategies in Australia and underscored the urgent need for proactive measures to address the complex health risks associated with climate change. It recommended undertaking a National Adaptation Plan process, improving understanding of managing health risks, and strengthening the public health system to build health resilience, particularly for vulnerable populations.<br>*Effectiveness:*<br>Inconclusive: No specific evaluation of the effectiveness of any intervention or adaptation measure.
Xu et al., 2023 [69]	*Study type:*<br>Scoping review<br>*Climate impact*:<br>Heatwaves, wildfires, and droughts	*Australian studies included:*<br>Not specified<br>*Setting:*<br>Not specified	*Aim(s):*<br>Illustrate spatiotemporal patterns of selected climate-related environmental extremes across Australia during the past two decades and summarise climate adaptation measures and actions that have been taken by the national, state/territory, and local governments.	*Intervention(s):*<br>Adaptation to heat, bushfires, floods and drought, climate-sensitive infectious diseases, impacts of climate change on Indigenous health.<br>*Outcome(s):*<br>Health effects, environmental indicators, capacity and capability of local workforce.	*Key finding(s):*<br>Significant impacts of climate-related environmental extremes on the health and well-being of Australians. Adaptation plans must be further developed to yield concrete actions.<br>*Effectiveness:*<br>Not assessed
Zurynski et al., 2024 [70]	*Study type:*<br>Systematic review<br>*Climate impact*:<br>Hurricanes, wildfires, floods	*Australian studies included:*<br>7<br>*Setting:*<br>Not specified	*Aim(s):*<br>Answer what are the health workforce impacts of climate-related events; and how are health systems preparing their workforces to respond to climate change impacts.	*Intervention(s):*<br>Responses to climate change-related events and preparations suggested to ensure the workforce is readily equipped for such events.<br>*Outcome(s):*<br>Preparedness, planning, upskilling and capacity building of health workforce.	*Key finding(s):*<br>Four impact themes were identified: (1) absenteeism; (2) psychological impacts; (3) system breakdown, and (4) unsafe working conditions.<br>Six responses and preparation themes: (1) training/skill development; (2) workforce capacity planning; (3) interdisciplinary collaboration; (4) role flexibility; (5) role incentivisation, and (6) psychological support.<br>*Effectiveness:*<br>Effective
Theme 3: Health co-benefits of climate change mitigation action outside the healthcare system					
Delany-Crowe et al., 2019 [71]	*Study type:*<br>Review (document analysis)<br>*Climate impact*:<br>Drought	*Australian studies included:*<br>178 strategic policies and Acts<br>*Setting:*<br>Not specified	*Aim(s):*<br>Analyse Australian policies on water management and climate change (are they supporting the sustainable development goals (SDGs) and improved health and	*Intervention(s):*<br>Water management.<br>*Outcome(s):*<br>SDGs on water, climate change, and marine ecosystems (Goals 6, 13 and 14).	*Key finding(s):*<br>A lack of comprehensive frameworks to address all drivers of climate change, and weaknesses in the management of waterways and marine ecosystems, still pose serious risks to the future of the natural environment and human well-being. Australian environmental risks were compounded

			well-being?)		by weak national leadership.<br>*Effectiveness:*<br>Not assessed
Giles-Corti et al., 2010 [72]	*Study type:*<br>Mini review<br>*Climate impact*:<br>Any	*Australian studies included:*<br>Not specified<br>*Setting:*<br>NA	*Aim(s):*<br>(1) What are the health benefits and co-benefits of investing in active transportation?<br>(2) How do different policy options which aim to create healthy, socially, and environmentally sustainable communities compare?	*Intervention(s):*<br>Active transportation.<br>*Outcome(s):*<br>(1) Health benefits; (2) social benefits; (3) reduced GHG emissions; (4) economic benefits (reduced healthcare cost).	*Key finding(s):*<br>Active transportation was associated with: (1) reduced premature mortality, improved respiratory health, reduced cardiovascular disease, obesity, and diabetes; (2) safer neighbourhoods, and reduction in public nuisance and crime, and marginalisation of vulnerable subgroups; and (3) reduction in traffic congestion and greenhouse gases emissions.<br>*Effectiveness:*<br>Not assessed
Lowe, 2014 [73]	*Study type:*<br>Review (informed by theoretical framework)<br>*Climate impact*:<br>Any	*Australian studies included:*<br>Not specified<br>*Setting:*<br>NA	*Aim(s):*<br>Provide an overview of the shared structural causes of obesity and climate change, and analyse policies that could be implemented in Australia to reduce obesity rates and contribute to climate change mitigation.	*Intervention(s):*<br>Policies with potential co-benefits for obesity and climate change mitigation, such as active transport and healthier diets.<br>*Outcome(s):*<br>Obesity outcomes and greenhouse gas emissions.	*Key finding(s):*<br>Complementary policies, including macro-level economic policies and more sector-specific policies, are required to achieve greenhouse gas emission reductions and equitable improvements across the transport, food and economic systems.<br>*Effectiveness:*<br>Significant public health, social and environmental co-benefits.
Willand et al., 2015 [74]	*Study type:*<br>Realist review<br>*Climate impact*:<br>Indoor heat stress	*Australian studies included:*<br>2<br>*Setting:*<br>Housing	*Aim(s):*<br>Explain the impacts of residential energy efficiency interventions on householder health, focusing on exploring pathways through which energy efficiency measures influence health outcomes, including cardiovascular, respiratory, mental, and social health; and to identify mediating factors, contextual issues, and the dynamic nature of outcomes related to energy efficiency improvements in households.	*Intervention(s):*<br>Housing interventions for warmth and energy efficiency, including thermal retrofits, upgrades, and comprehensive refurbishments to create warmer and drier living environments.<br>*Outcome(s):*<br>Health outcomes, indoor temperatures, humidity levels, and overall well-being of householders.	*Key finding(s):*<br>Interventions improving warmth and reducing humidity in winter were linked to benefits for cardiovascular and respiratory health. Positive effects on mental and social well-being were noted, often independent of energy cost savings, due to the enriched meaning of a comfortable home. While evidence of negative health impacts from inadequate ventilation was uncommon, it remains a concern that should not be overlooked. Ensuring warm homes during winter was identified as a critical factor for improving physiological, psychological, and social health outcomes.<br>*Effectiveness:*<br>Mixed: effectiveness evaluated based on various factors and while the interventions showed positive impacts, challenges in establishing clear associations between intervention categories and outcomes across the programs remain.
Theme 4: Adaptation and resilience interventions to protect health in sectors outside the healthcare system					
Adnan et al., 2022 [75]	*Study type:*<br>Systematic review<br>*Climate impact*:<br>Heat	*Australian studies included:*<br>107<br>*Setting:*<br>Urban	*Aim(s):*<br>Provide a systematic and overarching review of the different components of heatwave vulnerability (e.g., exposure, sensitivity, and adaptive capacity)	*Intervention(s):*<br>Urban heat exposure assessments, mitigation strategies, heat adaptation measures.<br>*Outcome(s):*	*Key finding(s):*<br>Green infrastructures are regarded as a sustainable intervention in mitigating heatwave impacts due to their multifaceted benefits. Extreme temperature impacts in various Australian cities can be reduced by adopting water sensitive urban design; the use of high albedo surface

			in Australia.	Reduced heat, heat-related morbidity, heat stress, thermal comfort, work-related injuries.	areas (as well as reflective and radiative cooling materials), when used at the local (building construction) scale, can increase the reflectance of solar radiation, and mitigate any potential heating effects. In terms of heat adaptation, thermal comfort-based urban planning can enhance heat adaptation. High temperature warning systems have already proven to be efficient in limiting occupational injuries in various Australian cities. *Effectiveness:* Not assessed
Charlson et al., 2021 [76]	*Study type:* Scoping review *Climate impact*: Heat, humidity, rainfall, drought, wildfires, and floods	*Australian studies included:* 34 *Setting:* Any	*Aim(s):* Assess the available literature related to climate change and mental health across the five global research priorities of the World Health Organization for protecting human health from climate change.	*Intervention(s):* Any interventions to mitigate or adapt to climate change. *Outcome(s):* Mental health and wellbeing.	*Key finding(s):* (1) A community development model, incorporating elements of health promotion, education, and early intervention, was considered effective in helping rural communities build capacity and resilience in the face of chronic drought-related hardship; (2) 90% of rural health service managers perceived climate change as likely to impact mental health and highlighted the important role of rural health services in education and advocacy on the health impacts of climate change; (3) community gardens improved social connectedness and mental and physical health. *Effectiveness:* Inconclusive
Desai & Zhang, 2021 [77]	*Study type:* Scoping review *Climate impact*: Floods, hurricanes, heat waves, droughts, poor air quality, water salinity, heavy snowfall, blizzards	*Australian studies included:* 3 *Setting:* Any	*Aim(s):* Better understand climate change and women's health (from a gender perspective) to support the development and implementation of climate change strategies and actions.	*Intervention(s):* Strategies to enhance local adaptive capacity to climate change, with more input from women's perspectives regarding management at household levels; Government assistance to women living in areas prone to extreme climatic effects, such as droughts. *Outcome(s):* Adaptive capacity, mitigating health impacts of climate change and strengthening resilience.	*Key finding(s):* Women are more negatively affected by droughts and heat waves due to their roles in society and nutritional and physiological requirements during periods of menstruation and pregnancy. Pregnant women are physically more vulnerable because of immune system changes due to hormonal alterations and are also sensitive to changes in temperatures. They are also more susceptible to infectious diseases and poor pregnancy outcomes. *Effectiveness:* Effective
Heaney et al., 2021 [78]	*Study type*: Scoping review *Climate impact*: Bushfire smoke	*Australian studies included:* 20 *Setting:* Community (urban, rural and remote)	*Aim(s):* Assess the evidence regarding optimal public communication strategies used in smoke-related disaster scenarios to inform the public health and emergency services on the best practices to connect with and empower populations to avoid exposure to bushfire smoke.	*Intervention(s):* Communication techniques utilised to disseminate health warnings to at-risk subgroups and the general population during bushfires and other natural disasters. *Outcome(s):* Smoke ($PM_{2.5}$)	*Key finding(s):* Social media, television, and radio are among the most common information sources utilised in bushfire smoke events. Message style, content, and method of delivery can directly influence message uptake and behaviour modification. Age, rurality, and geographical location influence information source preferences. *Effectiveness*: Not assessed

Harley et al., 2011 [79]	*Study type*: Narrative review *Climate impact*: Mosquito borne disease	*Australian studies included:* Not specified *Setting:* Population health	*Aim(s):* Current situation and potential future climate change impacts on respiratory, diarrheal, and vector-borne diseases in Australia.	*Intervention(s):* Health promotion and education, surveillance, early warning systems. *Outcome(s):* Impact of climate change on infectious diseases including respiratory, diarrheal, and vector-borne diseases.	*Key finding(s):* Decreased influenza and rotavirus incidence, but incidence of Salmonella was projected to increase in Australia. The significance of climate change effects on vector-borne diseases was disputed. *Effectiveness*: Not assessed
Jay et al., 2021 [80]	*Study type:* Narrative review *Climate impact*: Heat	*Australian studies included:* Not specified *Setting:* Global community	*Aim(s):* Describe how a future reliance on air conditioning is unsustainable and further marginalises the communities most vulnerable to the heat and show that a more holistic understanding of the thermal environment at the landscape and urban, building, and individual scales supports the identification of numerous sustainable opportunities to keep people cooler.	*Intervention(s):* Heat reduction strategies (from personal cooling strategies to green cities). *Outcome(s):* Temperature-related mortality and morbidity.	*Key finding(s):* (1) Evidence-based cooling strategies during heat extremes and hot weather are urgently needed to cope with the health risks associated with the inevitable trajectory of climate change; (2) air conditioning is set to become the most widely adopted heat reduction strategy worldwide, yet it is unaffordable for many of the most vulnerable, financially and environmentally costly, and leaves many defenceless from extreme heat during power outages; (3) strategies at the landscape, urban (e.g., blue and green spaces) and building (e.g., changing materials and natural ventilation) levels can greatly augment adaptive capacity to heat extremes and hot weather; (4) effective cooling solutions can be adopted at the individual level, even in low-resource settings, which are more sustainable than air conditioning, and focus on cooling the person to relieve physiological heat strain, as opposed to cooling the surrounding environment; (5) heat action plans that are robust, evidence-based, well communicated, and informed by real-time surveillance provide optimal health protection. *Effectiveness:* Urban ventilation pathways are especially effective when combined with blue and green infrastructure. Effectiveness of natural cross ventilation for existing buildings is dependent on orientation and window locations. Parks with elevated shading canopies are more effective urban climate moderators than unshaded grass-covered terrain. Self-dousing is effective up to at least 47°C. Reflective pavements and wall coatings are less effective due to the reflected solar radiation being absorbed by adjacent buildings and pedestrians.
Pitman et al., 2015 [81]	*Study type:* Systematic review *Climate impact*: Urban heat island effect (temperature),	*Australian studies included:* Not specified *Setting:* Urban environments and communities	*Aim(s):* Explore the role of Green Infrastructure in delivering multiple environmental, social, and economic values and services to urban communities.	*Intervention(s):* Green Infrastructure: strategic planning and implementation of green spaces and water systems within cities to provide multiple benefits, such as temperature reduction, improved	*Key finding(s):* By implementing strategic intervention measures and leveraging the benefits of Green Infrastructure, cities can better adapt to climate change challenges and create more sustainable and liveable environments for both humans and wildlife. *Effectiveness:*

	extreme weather events (floods, storms, extreme winds, and rainfall), climate change impacts on air quality and biodiversity.			air quality, enhanced biodiversity, and better water management.<br>*Outcome(s):*<br>Vegetation cover, tree planting, water management, stormwater harvesting, recycled water systems, micro-level wind management, and adiabatic cooling systems.	Inconclusive: no explicit mention of evaluation of effectiveness of Green Infrastructure. While the study emphasizes the benefits of Green Infrastructure, it primarily focuses on highlighting the advantages of Green Infrastructure rather than presenting a specific evaluation of its effectiveness.
Vien et al., 2024 [82]	*Study type:*<br>Scoping review<br>*Climate impact*:<br>Wildfire / bushfire	*Australian studies included:*<br>6<br>*Setting:*<br>Not specified	*Aim(s):*<br>Identify: (1) relevant peer-reviewed studies about wildfire smoke risk communications, including communication resources for vulnerable, at-risk populations; (2) characteristics of effective communications, dissemination strategies, and gaps in the peer-reviewed literature; and (3) recommendations to improve wildfire smoke research and communication practices.	*Intervention(s):*<br>Wildfire/bushfire smoke risk communications (communication materials, dissemination strategies, behaviour change, and communications for vulnerable audience).<br>*Outcome(s):*<br>Communication materials and messaging, and communication delivery strategies.	*Key finding(s):*<br>Limited studies describing behaviour change to reduce wildfire smoke exposure, characteristics of effective communication materials and messaging, and communication delivery strategies. Literature on risk communications, dissemination, and behaviour change for vulnerable populations was even more limited.<br>*Effectiveness:*<br>Inconclusive: many articles included some assessment of effectiveness of risk communications, but more work is needed.
Zhao et al., 2022 [83]	*Study type*:<br>Narrative review<br>*Climate impact*:<br>Suboptimal temperatures, wildfires, smoke, floods, droughts, water scarcity	*Australian studies included:*<br>5<br>*Setting:*<br>Not specified	*Aim(s):*<br>Review the effects of climate change on a broad range of health outcomes; to discuss mitigation and adaptation strategies against climate change and how these strategies may benefit human health in other ways.	*Intervention(s):*<br>Mitigation and adaptation solutions, including health adaptation plans and early warning systems.<br>*Outcome(s):*<br>Deaths, Emergency Department (ED) visits, hospital admissions, vector-borne diseases, diarrhoea incidence, mental health disorders	*Key finding(s):*<br>Pathways between climate change and human health, and possible solutions, including directions for future research.<br>*Effectiveness*:<br>Not assessed

AHS: Area Health Services, BCTs: Behaviour Change Techniques, CBD: Central Business District, CCA: Climate Change Adaptation, CO_2e: Carbon dioxide equivalent, COM-B: Capability, Opportunity, Motivation and Behaviour, CSAPHN: Country South Australia Primary Health Network, CT: Computerised Tomography, CVD: Cardiovascular disease, CXR: Chest X-ray, DHT: Digital Health Technologies, dRM: Repeated measures effect size estimates, DSM-5: Diagnostic and Statistical Manual of Mental Disorders, Fifth Edition, ED: Emergency Department, EHR: Electronic health records, GHG: Greenhouse gases, GMR: Greater Metropolitan Region, GWP100: Global warming potential of a greenhouse gas relative to carbon dioxide over a 100-year period, HAP: Heat Action Plans, HCW: Health Care Worker, HEPA: High Efficiency Particulate Air, HHI: Heat Health Impact, HHWS: Heat-Health Warning System, HIA: Health Impact Assessment, HWS: Heatwave Warning System, ICT: Information Communication Technology, ILM: Indigenous Land Management, ISLHD: Illawarra Shoalhaven Local Health District, IV: Intravenous, LMA: Laryngeal mask airway, MCXR: Mobile Chest X-ray, MGP: Midwifery group practice, MHFA: Mental Health First Aid, MJA: Medical Journal of Australia, MRI: Magnetic Resonance Imaging,

NA: Not Applicable, NHN: Northern Health Network, NHS: National Health Service, OR: Operating Room, PCL-5: PTSD Checklist for DSM-5, PPE: Personal Protective Equipment, PSYCHLOPS: Psychological Outcome Profiles instrument, PTSD: Post-Traumatic Stress Disorder, QI: Quality improvement, RAMHP: Rural Adversity Mental Health Program, RRV: Ross River Virus, SC: Standard care, SDG: Sustainable Development Goals, SOLAR: Skills fOr Life Adjustment and Resilience, TIVA: Total Intravenous Anaesthesia, UCE: Urban Cooling Effect, UHMT: Urban Heat Mitigation Techniques, US: Ultrasound, UTCI: Universal Thermal Climate Index, WHS: Work Health and Safety

Preferred Reporting Items for Systematic reviews and Meta-Analyses extension for Scoping Reviews (PRISMA-ScR) Checklist

SECTION	ITEM	PRISMA-ScR CHECKLIST ITEM	REPORTED ON PAGE #
**TITLE**			
Title	1	Identify the report as a scoping review.	The manuscript has been identified as a systematic mapping review
**ABSTRACT**			
Structured summary	2	Provide a structured summary that includes (as applicable): background, objectives, eligibility criteria, sources of evidence, charting methods, results, and conclusions that relate to the review questions and objectives.	A structured abstract has been provided
**INTRODUCTION**			
Rationale	3	Describe the rationale for the review in the context of what is already known. Explain why the review questions/objectives lend themselves to a scoping review approach.	Rationale provided in Introduction
Objectives	4	Provide an explicit statement of the questions and objectives being addressed with reference to their key elements (e.g., population or participants, concepts, and context) or other relevant key elements used to conceptualize the review questions and/or objectives.	The objectives and aim of the review have been articulated in the abstract and introduction
**METHODS**			
Protocol and registration	5	Indicate whether a review protocol exists; state if and where it can be accessed (e.g., a Web address); and if available, provide registration information, including the registration number.	PROSPERO registration is not possible for systematic mapping reviews
Eligibility criteria	6	Specify characteristics of the sources of evidence used as eligibility criteria (e.g., years considered, language, and publication status), and provide a rationale.	Eligibility criteria provided in Supporting Information
Information sources*	7	Describe all information sources in the search (e.g., databases with dates of coverage and contact with authors to identify additional sources), as well as the date the most recent search was executed.	Databases and dates of coverage provided in Methods
Search	8	Present the full electronic search strategy for at least 1 database, including any limits used, such that it could be repeated.	Full search strategy provided in Supporting Information
Selection of sources of evidence†	9	State the process for selecting sources of evidence (i.e., screening and eligibility) included in the scoping review.	Process for selecting sources of evidence described in Methods
Data charting process‡	10	Describe the methods of charting data from the included sources of evidence (e.g., calibrated forms or forms that have been tested by the team before their use, and whether data charting was done independently or in duplicate) and any processes for obtaining and confirming data from investigators.	The process for extracting and thematically mapping data is described in Methods
Data items	11	List and define all variables for which data were sought and any assumptions and simplifications made.	Data variables listed in Methods
Critical appraisal of individual sources of evidence§	12	If done, provide a rationale for conducting a critical appraisal of included sources of evidence; describe the methods used and how this information was used in any data synthesis (if appropriate).	Critical appraisal conducted to highlight the strengths and weaknesses of the evidence base
Synthesis of results	13	Describe the methods of handling and summarizing the data that were charted.	Evidence was summarized under four pre-defined themes
**RESULTS**			
Selection of sources of evidence	14	Give numbers of sources of evidence screened, assessed for eligibility, and included in the review, with reasons for exclusions at each stage, ideally using a flow diagram.	PRISMA diagram provided in Methods
Characteristics of	15	For each source of evidence, present characteristics for	Characteristics for which

SECTION	ITEM	PRISMA-ScR CHECKLIST ITEM	REPORTED ON PAGE #
sources of evidence		which data were charted and provide the citations.	data were mapped are presented in Results with citations
Critical appraisal within sources of evidence	16	If done, present data on critical appraisal of included sources of evidence (see item 12).	Data on critical appraisal presented in Results
Results of individual sources of evidence	17	For each included source of evidence, present the relevant data that were charted that relate to the review questions and objectives.	Extracted data are presented under each theme
Synthesis of results	18	Summarize and/or present the charting results as they relate to the review questions and objectives.	Results and recommendations provided
**DISCUSSION**			
Summary of evidence	19	Summarize the main results (including an overview of concepts, themes, and types of evidence available), link to the review questions and objectives, and consider the relevance to key groups.	Main results summarized in Discussion
Limitations	20	Discuss the limitations of the scoping review process.	Limitations discussed in separate sub-section
Conclusions	21	Provide a general interpretation of the results with respect to the review questions and objectives, as well as potential implications and/or next steps.	Overall interpretation of the results and potential implications provided
**FUNDING**			
Funding	22	Describe sources of funding for the included sources of evidence, as well as sources of funding for the scoping review. Describe the role of the funders of the scoping review.	Sources of funding and role of funders provided

JBI = Joanna Briggs Institute; PRISMA-ScR = Preferred Reporting Items for Systematic reviews and Meta-Analyses extension for Scoping Reviews.

* Where *sources of evidence* (see second footnote) are compiled from, such as bibliographic databases, social media platforms, and Web sites.

† A more inclusive/heterogeneous term used to account for the different types of evidence or data sources (e.g., quantitative and/or qualitative research, expert opinion, and policy documents) that may be eligible in a scoping review as opposed to only studies. This is not to be confused with *information sources* (see first footnote).

‡ The frameworks by Arksey and O'Malley (6) and Levac and colleagues (7) and the JBI guidance (4, 5) refer to the process of data extraction in a scoping review as data charting.

§ The process of systematically examining research evidence to assess its validity, results, and relevance before using it to inform a decision. This term is used for items 12 and 19 instead of "risk of bias" (which is more applicable to systematic reviews of interventions) to include and acknowledge the various sources of evidence that may be used in a scoping review (e.g., quantitative and/or qualitative research, expert opinion, and policy document).

From: Tricco AC, Lillie E, Zarin W, O'Brien KK, Colquhoun H, Levac D, et al. PRISMA Extension for Scoping Reviews (PRISMAScR): Checklist and Explanation. Ann Intern Med. 2018;169:467–473. doi: 10.7326/M18-0850.

References

1. Breth-Petersen M, Barratt AL, McGain F, Skowno JJ, Zhong G, Weatherall AD, Bell KJ, Pickles KM. Exploring anaesthetists' views on the carbon footprint of anaesthesia and identifying opportunities and challenges for reducing its impact on the environment. Anaesthesia and Intensive Care. 2024;52:91-104.
2. Charlesworth KE, Jamieson M. Healthcare in a carbon-constrained world. Australian Health Review. 2019;43:241-245.
3. Davies JF, McAlister S, Eckelman MJ, McGain F, Seglenieks R, Gutman EN, Groome J, Palipane N, Latoff K, Nielsen D, Sherman JD; TRA2SH, GASP and WAAREN collaborators. Environmental and financial impacts of perioperative paracetamol use: a multicentre international life-cycle analysis. British Journal of Anaesthesia. 2024;133:1439-1448.
4. Davis NF, McGrath S, Quinlan M, Jack G, Lawrentschuk N, Bolton DM. Carbon footprint in flexible ureteroscopy: A comparative study on the environmental impact of reusable and single-use ureteroscopes. Journal of Endourology. 2018;32:214-217.
5. Ellis I, Cheek C, Jaffray L, Skinner T. Making a case for telehealth: measuring the carbon cost of health-related travel. Rural and Remote Health. 2013;13:2723.
6. McAlister S, Barratt AL, Bell KJL, McGain F. The carbon footprint of pathology testing. Medical Journal of Australia. 2020;212:377-382.
7. McAlister S, McGain F, Petersen M, Story D, Charlesworth K, Ison G, Barratt A. The carbon footprint of hospital diagnostic imaging in Australia. The Lancet Regional Health – Western Pacific. 2022;24:100459.
8. McAlister S, Smyth B, Koprivic I, Luca Di Tanna G, McGain F, Charlesworth K, Brown MA, Konecny P. Carbon emissions and hospital pathology stewardship: A retrospective cohort analysis. Internal Medicine Journal. 2023;53:584-589.
9. McGain F, McAlister S, McGavin A, Story D. The financial and environmental costs of reusable and single-use plastic anaesthetic drug trays. Anaesthesia and Intensive Care. 2010;38:538-544.
10. McGain F, McAlister S, McGavin A, Story D. A life cycle assessment of reusable and single-use central venous catheter insertion kits. Anesthesia and Analgesia. 2012;114(5):1073-1080.
11. McGain F, Story D, Lim T, McAlister S. Financial and environmental costs of reusable and single-use anaesthetic equipment. British Journal of Anaesthesia. 2017;118:862-869.
12. Talbot B, Barraclough K, Sypek M, Gois P, Arnold L, McDonald S, Knight J. A survey of environmental sustainability practices in dialysis facilities in Australia and New Zealand. Clinical Journal of the American Society of Nephrology. 2022;17:1792-1799.
13. Wyssusek K, Chan KL, Eames G, Whately Y. Greenhouse gas reduction in anaesthesia practice: a departmental environmental strategy. BMJ Open Quality. 2022;11:e001867.

14. de Souza M, Lee AB, Cook S. Healthy patients, workforce and environment: Coupling climate adaptation and mitigation to wellbeing in healthcare. International Journal of Environmental Research and Public Health. 2023;20:7059.
15. Kildea S, Simcock G, Liu A, Elgbeili G, Laplante DP, Kahler A, Austin MP, Tracy S, Kruske S, Tracy M, O'Hara MW, King S. Continuity of midwifery carer moderates the effects of prenatal maternal stress on postnatal maternal wellbeing: the Queensland flood study. Archives of Women's Mental Health. 2018;21:203-214.
16. Knezevic A, Olcoń K, Smith L, Allan J, Pai P. Wellness Warriors: a qualitative exploration of healthcare staff learning to support their colleagues in the aftermath of the Australian bushfires. International Journal of Qualitative Studies on Health and Well-being. 2023;18:2167298.
17. Marfori MT, Campbell SL, Garvey K, McKeown S, Veitch M, Wheeler AJ, Borchers-Arriagada N, Johnston FH. Public health messaging during extreme smoke events: Are we hitting the mark? Frontiers in Public Health. 2020;8:465.
18. McLean M, Phelps C, Smith J, Maheshwari N, Veer V, Bushell D, Matthews R, Craig B, Moro C. An authentic learner-centered planetary health assignment: A five-year evaluation of student choices to address Sustainable Development Goal 13 (Climate Action). Frontiers in Public Health. 2022;10:1049932.
19. Mohtady Ali H, Ranse J, Roiko A, Desha C. Healthcare workers’ resilience toolkit for disaster management and climate change adaptation. International Journal of Environmental Research and Public Health. 2022;19:12440.
20. Nitschke M, Krackowizer A, Hansen AL, Bi P, Tucker GR. Heat health messages: A randomized controlled trial of a preventative messages tool in the older population of South Australia. International Journal of Environmental Research and Public Health. 2017;14:992.
21. Nitschke M, Tucker G, Hansen A, Williams S, Zhang Y, Bi P. Evaluation of a heat warning system in Adelaide, South Australia, using case-series analysis. BMJ Open. 2016;6:e012125.
22. Patrick R, Capetola T. It's here! Are we ready? Five case studies of health promotion practices that address climate change from within Victorian health care settings. Health Promotion Journal of Australia. 2011;22:S61-S67.
23. Patrick R, Kingsley J. Health promotion and sustainability programmes in Australia: barriers and enablers to evaluation. Global Health Promotion. 2019;26:82-92.
24. van Beurden EK, Kia AM, Hughes D, Fuller JD, Dietrich U, Howton K, Kavooru S. Networked resilience in rural Australia – a role for health promotion in regional responses to climate change. Health Promotion Journal of Australia. 2011;22:S54-S60.
25. Walker R, South East Healthy Communities Partnership. Climate change and primary health care intervention framework. Australian Journal of Primary Health. 2009;15:276-284.

26. Williams S, Hanson-Easey S, Nitschke M, Howell S, Nairn J, Beattie C, Wynwood G, Bi P. Heat-health warnings in regional Australia: examining public perceptions and responses. Environmental Hazards. 2019;18:287-310.
27. Williams S, Nitschke M, Wondmagegn BY, Tong M, Xiang J, Hansen A, Nairn J, Karnon J, Bi P. Evaluating cost benefits from a heat health warning system in Adelaide, South Australia. Australian and New Zealand Journal of Public Health. 2022;46:149-154.
28. Burgess CP, Johnston FH, Berry HL, McDonnell J, Yibarbuk D, Gunabarra C, Mileran A, Bailie RS. Healthy country, healthy people: the relationship between Indigenous health status and "caring for country". Medical Journal of Australia. 2009;190:567-572.
29. Haddad S, Paolini R, Ulpiani G, Synnefa A, Hatvani-Kovacs G, Garshasbi S, Fox J, Vasilakopoulou K, Nield L, Santamouris M. Holistic approach to assess co-benefits of local climate mitigation in a hot humid region of Australia. Scientific Reports. 2020;10:14216.
30. Ridoutt B, Baird D, Hendrie GA. Diets within environmental limits: The climate impact of current and recommended Australian diets. Nutrients. 2021;13:1122.
31. Schultz R, Abbott T, Yamaguchi J, Cairney S. Indigenous land management as primary health care: qualitative analysis from the Interplay research project in remote Australia. BMC Health Services Research. 2018;18:960.
32. Springmann M, Sacks G, Ananthapavan J, Scarborough P. Carbon pricing of food in Australia: an analysis of the health, environmental and public finance impacts. Australian and New Zealand Journal of Public Health. 2018;42:523-529.
33. Chen D, Wang X, Thatcher M, Barnett G, Kachenko A, Prince R. Urban vegetation for reducing heat related mortality. Environmental Pollution. 2014;192:275-284.
34. Cowlishaw S, Gibson K, Alexander S, Howard A, Agathos J, Strauven S, Chisholm K, Fredrickson J, Pham L, Lau W, O'Donnell ML. Improving mental health following multiple disasters in Australia: a randomized controlled trial of the Skills for Life Adjustment and Resilience (SOLAR) programme. European Journal of Psychotraumatology. 2023;14:2284032.
35. Dufty N. Using heat refuges in heatwave emergencies. Australian Journal of Emergency Management. 2022;36:38-44.
36. Hansen A, Bi P, Nitschke M, Pisaniello D, Newbury J, Kitson A. Perceptions of heat-susceptibility in older persons: barriers to adaptation. International Journal of Environmental Research and Public Health. 2011;8:4714-4728.
37. Hart CR, Berry HL, Tonna AM. Improving the mental health of rural New South Wales communities facing drought and other adversities. Australian Journal of Rural Health. 2011;19:231-238.
38. Longman J, Braddon M, Verlie B, Schlosberg D, Hampshire L, Hawke C, Noonan A, Saurman E. Building resilience to the mental health impacts of climate change in rural Australia. The Journal of Climate Change and Health. 2023;12:100240.

39. McGill N, Curtin M, Hodgins G, Parnell T, Verdon S, Crockett J, Davison WR. Supporting children's recovery from bushfires: Stakeholders' views about the impact of a community-based intervention program on children. Australian Journal of Rural Health. 2024;32:42-52.
40. O'Donnell ML, Lau W, Fredrickson J, Gibson K, Bryant RA, Bisson J, Burke S, Busuttil W, Coghlan A, Creamer M, Gray D, Greenberg N, McDermott B, McFarlane AC, Monson CM, Phelps A, Ruzek JI, Schnurr PP, Ugsang J, Watson P, Whitton S, Williams R, Cowlishaw S, Forbes D. An open label pilot study of a brief psychosocial intervention for disaster and trauma survivors. Frontiers in Psychiatry. 2020;11:483.
41. Qi J, Ding L, Lim S. Toward cool cities and communities: A sensitivity analysis method to identify the key planning and design variables for urban heat mitigation techniques. Sustainable Cities and Society. 2021;75:103377.
42. Quilty S, Jupurrurla NF, Lal A, Matthews V, Gasparrini A, Hope P, Brearley M, Ebi KL. The relative value of sociocultural and infrastructural adaptations to heat in a very hot climate in northern Australia: a case time series of heat-associated mortality. The Lancet Planetary Health. 2023;7:e684-e693.
43. Rigby CW, Rosen A, Berry HL, Hart CR. If the land's sick, we're sick:* the impact of prolonged drought on the social and emotional well-being of Aboriginal communities in rural New South Wales. Australian Journal of Rural Health. 2011;19:249-254.
44. Sadeghi M, Chaston T, Hanigan I, de Dear R, Santamouris M, Jalaludin B, Morgan GG. The health benefits of greening strategies to cool urban environments – A heat health impact method. Building and Environment. 2022;207:108546.
45. Santamouris M, Paolini R, Haddad S, Synnefa A, Garshasbi S, Hatvani-Kovacs G, Gobakis K, Yenneti K, Vasilakopoulou K, Feng J, Gao K, Papangelis G, Dandou A, Methymaki G, Portalakis P, Tombrou M. Heat mitigation technologies can improve sustainability in cities. An holistic experimental and numerical impact assessment of urban overheating and related heat mitigation strategies on energy consumption, indoor comfort, vulnerability and heat-related mortality and morbidity in cities. Energy and Buildings. 2020;217:110002.
46. Seale H, Trent M, Marks GB, Shah S, Chughtai AA, MacIntyre CR. Exploring the use of masks for protection against the effects of wildfire smoke among people with preexisting respiratory conditions. BMC Public Health. 2023;23:2330.
47. Tomerini DM, Dale PE, Sipe N. Does mosquito control have an effect on mosquito-borne disease? The case of Ross River virus disease and mosquito management in Queensland, Australia. Journal of the American Mosquito Control Association. 2011;27:39-44.
48. Varghese BM, Hansen AL, Williams S, Bi P, Hanson-Easey S, Barnett AG, Heyworth JS, Sim MR, Rowett S, Nitschke M, Di Corleto R, Pisaniello DL. Heat-related injuries in Australian workplaces: Perspectives from health and safety representatives. Safety Science. 2020;126:104651.

49. Wheeler AJ, Allen RW, Lawrence K, Roulston CT, Powell J, Williamson GJ, Jones PJ, Reisen F, Morgan GG, Johnston FH. Can public spaces effectively be used as cleaner indoor air shelters during extreme smoke events? International Journal of Environmental Research and Public Health. 2021;18:4085.
50. Zhang Y, Beggs PJ, Bambrick H, Berry HL, Linnenluecke MK, Trueck S, Alders R, Bi P, Boylan SM, Green D, Guo Y, Hanigan IC, Hanna EG, Malik A, Morgan GG, Stevenson M, Tong S, Watts N, Capon AG. The MJA-Lancet countdown on health and climate change: Australian policy inaction threatens lives. Medical Journal of Australia. 2018;209:474.
51. Beggs PJ, Zhang Y, Bambrick H, Berry HL, Linnenluecke MK, Trueck S, Bi P, Boylan SM, Green D, Guo Y, Hanigan IC, Johnston FH, Madden DL, Malik A, Morgan GG, Perkins-Kirkpatrick S, Rychetnik L, Stevenson M, Watts N, Capon AG. The 2019 report of the MJA–Lancet countdown on health and climate change: a turbulent year with mixed progress. Medical Journal of Australia. 2019;211:490-491.e21.
52. Zhang Y, Beggs PJ, McGushin A, Bambrick H, Trueck S, Hanigan IC, Morgan GG, Berry HL, Linnenluecke MK, Johnston FH, Capon AG, Watts N. The 2020 special report of the MJA–Lancet countdown on health and climate change: lessons learnt from Australia’s “Black Summer”. Medical Journal of Australia. 2020;213:490-492.e10.
53. Beggs PJ, Zhang Y, McGushin A, Trueck S, Linnenluecke MK, Bambrick H, Berry HL, Jay O, Rychetnik L, Hanigan IC, Morgan GG, Guo Y, Malik A, Stevenson M, Green D, Johnston FH, McMichael C, Hamilton I, Capon AG. The 2021 report of the MJA–Lancet countdown on health and climate change: Australia increasingly out on a limb. Medical Journal of Australia. 2021;215:390-392.e22.
54. Beggs PJ, Zhang Y, McGushin A, Trueck S, Linnenluecke MK, Bambrick H, Capon AG, Vardoulakis S, Green D, Malik A, Jay O, Heenan M, Hanigan IC, Friel S, Stevenson M, Johnston FH, McMichael C, Charlson F, Woodward AJ, Romanello MB. The 2022 report of the MJA-Lancet countdown on health and climate change: Australia unprepared and paying the price. Medical Journal of Australia. 2022;217:439-458.
55. Beggs PJ, Trueck S, Linnenluecke MK, Bambrick H, Capon AG, Hanigan IC, Arriagada NB, Cross TJ, Friel S, Green D, Heenan M, Jay O, Kennard H, Malik A, McMichael C, Stevenson M, Vardoulakis S, Dang TN, Garvey G, Lovett R, Matthews V, Phung D, Woodward AJ, Romanello MB, Zhang Y. The 2023 report of the MJA–Lancet countdown on health and climate change: sustainability needed in Australia's health care sector. Medical Journal of Australia. 2024;220:282-303.
56. Duindam D. Transitioning to sustainable healthcare: Decarbonising healthcare clinics, a literature review. Challenges. 2022;13:68.
57. Liu Y, Lee-Archer P, Sheridan NM, Seglenieks R, McGain F, Eley VA. Nitrous oxide use in Australian health care: Strategies to reduce the climate impact. Anesthesia and Analgesia. 2023;137:819-829.
58. McGain F, Naylor C. Environmental sustainability in hospitals – a systematic review and research agenda. Journal of Health Services Research and Policy. 2014;19:245-252.
59. Pencheon D, Rissel CE, Hadfield G, Madden DL. Health sector leadership in mitigating climate change: experience from the UK and NSW. New South Wales Public Health Bulletin. 2009;20:173-176.

60. Wyssusek KH, Keys MT, van Zundert AAJ. Operating room greening initiatives - the old, the new, and the way forward: A narrative review. Waste Management and Research. 2019;37:3-19.
61. Blashki G, Armstrong G, Berry HL, Weaver HJ, Hanna EG, Bi P, Harley D, Spickett JT. Preparing health services for climate change in Australia. Asia Pacific Journal of Public Health. 2011;23:133S-143S.
62. Crandon TJ, Dey C, Scott JG, Thomas HJ, Ali S, Charlson FJ. The clinical implications of climate change for mental health. Nature Human Behaviour. 2022;6:1474-1481.
63. Hu Y, Cheng J, Liu S, Tan J, Yan C, Yu G, Yin Y, Tong S. Evaluation of climate change adaptation measures for childhood asthma: A systematic review of epidemiological evidence. Science of The Total Environment. 2022;839:156291.
64. Lokmic-Tomkins Z, Bhandari D, Bain C, Borda A, Kariotis TC, Reser D. Lessons learned from natural disasters around digital health technologies and delivering quality healthcare. International Journal of Environmental Research and Public Health. 2023;20:4542.
65. Palinkas LA, O'Donnell ML, Lau W, Wong M. Strategies for delivering mental health services in response to global climate change: A narrative review. International Journal of Environmental Research and Public Health. 2020;17:8562.
66. Vu A, Rutherford S, Phung D. Heat health prevention measures and adaptation in older populations - A systematic review. International Journal of Environmental Research and Public Health. 2019;16:4370.
67. Walker R, Hassall J, Chaplin S, Congues J, Bajayo R, Mason W. Health promotion interventions to address climate change using a primary health care approach: a literature review. Health Promotion Journal of Australia. 2011;22:S6-S12.
68. Walter TG, Bricknell LK, Preston RG, Crawford EGC. Climate change adaptation methods for public health prevention in Australia: an integrative review. Current Environmental Health Reports. 2024;11:71-87.
69. Xu R, Yu P, Liu Y, Chen G, Yang Z, Zhang Y, Wu Y, Beggs PJ, Zhang Y, Boocock J, Ji F, Hanigan I, Jay O, Bi P, Vargas N, Leder K, Green D, Quail K, Huxley R, Jalaludin B, Hu W, Dennekamp M, Vardoulakis S, Bone A, Abrahams J, Johnston FH, Broome R, Capon T, Li S, Guo Y. Climate change, environmental extremes, and human health in Australia: challenges, adaptation strategies, and policy gaps. The Lancet Regional Health – Western Pacific. 2023;40:100936.
70. Zurynski Y, Fisher G, Wijekulasuriya S, Leask E, Dharmayani PNA, Ellis LA, Smith CL, Braithwaite J. Bolstering health systems to cope with the impacts of climate change events: A review of the evidence on workforce planning, upskilling, and capacity building. The International Journal of Health Planning and Management. 2024;39:781-805.
71. Delany-Crowe T, Marinova D, Fisher M, McGreevy M, Baum F. Australian policies on water management and climate change: are they supporting the sustainable development goals and improved health and well-being? Global Health. 2019;15:68.
72. Giles-Corti B, Foster S, Shilton T, Falconer R. The co-benefits for health of investing in active transportation. New South Wales Public Health Bulletin. 2010;21:122-127.

73. Lowe M. Obesity and climate change mitigation in Australia: overview and analysis of policies with co-benefits. Australian and New Zealand Journal of Public Health. 2014;38:19-24.
74. Willand N, Ridley I, Maller C. Towards explaining the health impacts of residential energy efficiency interventions - A realist review. Part 1: Pathways. Social Science and Medicine. 2015;133:191-201.
75. Adnan MSG, Dewan A, Botje D, Shahid S, Hassan QK. Vulnerability of Australia to heatwaves: A systematic review on influencing factors, impacts, and mitigation options. Environmental Research. 2022;213:113703.
76. Charlson F, Ali S, Benmarhnia T, Pearl M, Massazza A, Augustinavicius J, Scott JG. Climate change and mental health: A scoping review. International Journal of Environmental Research and Public Health. 2021;18:4486.
77. Desai Z, Zhang Y. Climate change and women's health: A scoping review. GeoHealth. 2021;5:e2021GH000386.
78. Heaney E, Hunter L, Clulow A, Bowles D, Vardoulakis S. Efficacy of communication techniques and health outcomes of bushfire smoke exposure: A scoping review. International Journal of Environmental Research and Public Health. 2021;18:10889.
79. Harley D, Bi P, Hall G, Swaminathan A, Tong S, Williams C. Climate change and infectious diseases in Australia: future prospects, adaptation options, and research priorities. Asia Pacific Journal of Public Health. 2011;23:54S-66S.
80. Jay O, Capon A, Berry P, Broderick C, de Dear R, Havenith G, Honda Y, Kovats RS, Ma W, Malik A, Morris NB, Nybo L, Seneviratne SI, Vanos J, Ebi KL. Reducing the health effects of hot weather and heat extremes: from personal cooling strategies to green cities. The Lancet. 2021;398:709-724.
81. Pitman SD, Daniels CB, Ely ME. Green infrastructure as life support: Urban nature and climate change. Transactions of the Royal Society of South Australia. 2015;139:97-112.
82. Vien MH, Ivey SL, Boyden H, Holm S, Neuhauser L. A scoping review of wildfire smoke risk communications: issues, gaps, and recommendations. BMC Public Health. 2024;24:312.
83. Zhao Q, Yu P, Mahendran R, Huang W, Gao Y, Yang Z, Ye T, Wen B, Wu Y, Li S, Guo Y. Global climate change and human health: Pathways and possible solutions. Eco-Environment and Health. 2022;1:53-62.
